# Primary Health Professionals’ Beliefs, Experiences, and Willingness to Treat Minor-Attracted Persons

**DOI:** 10.1007/s10508-021-02271-7

**Published:** 2022-01-27

**Authors:** Rebecca Lievesley, Helen Swaby, Craig A. Harper, Ellie Woodward

**Affiliations:** 1 Department of Psychology, Nottingham Trent University, 50 Shakespeare Street, Nottingham, NG1 4FQ UK; 2 Department of Counselling, Bishop Grosseteste University, Lincoln, UK

**Keywords:** Minor-attracted persons, Healthcare professionals, Treatment, Stigma, Pedophilia, DSM-5

## Abstract

There is a desire and need among minor-attracted persons (MAPs) to access support within the community, and this often begins with an approach to healthcare providers working in general medical/mental health settings. However, little is known about the experiences of these non-specialist professionals in relation to their beliefs, knowledge, and decision-making processes when working with patients who disclose sexual attractions to children. Using an online survey, this study explored the knowledge, comfort, competence, and treatment willingness of 220 non-specialist healthcare providers when faced with patients who disclose sexual attractions to children. We investigated how often such disclosures were made, clinician stigma, treatment priorities, and professionals’ willingness to report MAPs to external agencies because of their sexual attractions. Some key differences were found when comparing primary medical vs. mental health professionals, including increased likelihood to view MAPs as dangerous, unable to control behaviors and that sexual attractions are an avoidable choice, in the former group. Both groups prioritized mental health treatment targets above controlling attractions and living with stigmatized attractions, although controlling or changing attractions were still relatively high priorities. Results indicated a need for further training, focusing on increasing comfort around working with MAPs, as this was associated with a greater willingness to work with this group. We identify current gaps in service provision for MAPs seeking professional support and discuss recommendations for professional training.

**Supplementary Information:**

The online version contains supplementary material available at 10.1007/s10508-021-02271-7.

## Introduction

There is a growing body of research seeking to better understand and support individuals with a sexual attraction toward children (Cantor & McPhail, 2016; Grady et al., 2019; Houtepen et al., 2016; Jahnke et al., 2015a, 2015b, 2018; Levenson & Grady, 2019b; Lievesley et al., 2020; Stevens & Wood, 2019). Minor-attracted persons (MAPs) express sexual attractions to children that can be classified into an age-graded theory of several chronophilic categories (Seto, 2017). Pedophilia is the most widely studied of the chronophilias (Blanchard et al., 2009; Cantor et al., 2004, 2008, 2015; Seto, 2009, 2012; Tenbergen et al., 2015) and is defined as a primary or predominant sexual attraction to prepubescent children typically aged between 3 and 10 years (Blanchard et al., 2009; Seto, 2017). Other minor-related chronophilic categories include nepiophilia (sexual attractions to young infants), hebephilia (sexual attractions to pubescent children), and ephebophilia (sexual attractions to older teenagers who are typically between the ages of 15–17 years). Owing to controversy over the latter of these categories (Stephens & Seto, 2018), we use the broader MAP label to refer to individuals who have sexual attractions to infants, prepubescent children, and children who are in the early stages of puberty, due to the substantial self-reported and clinically observed overlap in these attraction patterns (Beier et al., 2009; Blanchard, 2010; Lievesley et al., 2020; Stephens et al., 2017a, b, 2018, 2019). In our case, we do not necessarily equate MAPs with those who have primary or predominant sexual attractions to children, but instead this label includes anybody who experiences such attractions, owing to the often observed lack of exclusivity of sexual attractions to children among MAPs (Bailey et al., 2016; Eher et al., 2015; Lievesley & Harper, 2021; Martijn et al., 2020).

As will be explored, there is a desire and need among MAPs to access support within the community for several reasons. According to surveys of MAPs (B4U-ACT, 2011), the primary focus of this population when seeking support is related to coping with mental health issues that stem from a combination of social and self-stigmatization (Goodier & Lievesley, 2018; Grady et al., 2019; Jahnke, 2018; Levenson & Grady, 2019b; Lievesley & Harper, 2021; McPhail et al., 2018). As such, in this paper we present what we believe to be the first systematic analysis of the beliefs, knowledge, and decision-making processes of primary (i.e., non-specialist) healthcare professionals in relation to working with patients who disclose sexual attractions to children. Although other work has predominantly explored the views of psychological professionals and specialists working in the field of sexual abuse prevention (Beggs Christofferson, 2019; Goodier & Lievesley, 2018; Levenson & Grady, 2019a; Parr & Pearson, 2019; Stephens et al., 2021), or students about to emerge as professionals in social work or psychotherapy (Walker et al., 2022), here we focus on those who work in primary healthcare with no specialism in MAP treatment or the prevention of sexual abuse. This is important as we know that many MAPs approach their family doctor or a private psychotherapist about related issues before accessing services that are specifically targeted at them (Levenson et al., 2020). As such, our analysis here provides a snapshot of the kinds of views that MAPs may be faced with when first accessing attraction-related support within primary healthcare settings.

### Help-Seeking in MAPs

There are two broad motivations acknowledged within the existing literature as to why MAPs might engage in help-seeking behaviors with reference to their sexual attractions. The first, and most widely endorsed within the literature, is focused on the prevention of child sexual abuse. Although many MAPs will never commit sexual offenses against children (Cantor & McPhail, 2016), pedophilia (as a specific form of minor attraction) has been documented as an important motivator of sexual offending (Finkelhor, 1984; Seto, 2009, 2019; Ward & Siegert, 2002). It has also been found to be a consistent predictor of sexual recidivism among individuals with sexual convictions (Stephens et al., 2017a, b). Despite this, it is important to distinguish between pedophilia (and other child-directed chronophilias) as a sexual attraction pattern, from sexual offending as a behavior. Although a relationship between attraction and behavior does seem to exist when studied within convicted samples, research suggests that only around half of all individuals with convictions for child sexual offenses meet the clinical criteria for being designated as having predominant or exclusive sexual attractions to children (i.e., meeting the clinical criteria for a designation of pedophilia; Schmidt et al., 2013). Although the literature highlights the benefits of early therapeutic support in prevention-oriented settings (Allardyce, 2018; Hocken, 2018; Lievesley & Harper, 2021), the most prominent program—the Dunkelfeld Project based in Berlin, Germany—has not been found to produce statistically significant reductions in dynamic risk indices for sexual offending (Mokros & Banse, 2019). This lack of definitive evidence of effectiveness may be due to the preliminary nature of early efficacy analyses, and as such further work is needed to truly evaluate the success of such targeted initiatives.

In response to MAP narratives about their own treatment targets and the lack of current empirical support for prevention-focused initiatives, some authors have shifted focus to highlight the importance of wellbeing-related support services for MAPs, which represents the second acknowledged motivation for MAPs’ help-seeking behaviors (B4U-ACT, 2011; Goodier & Lievesley, 2018; Grady et al., 2019; Levenson & Grady, 2019b; Lievesley & Harper, 2021; Lievesley et al., 2018). Such an approach appears to be more responsive to MAPs’ own treatment desires (B4U-ACT, 2011) and is in line with the current empirical assumption that enduring sexual attractions to children are largely unchangeable (Grundmann et al., 2016; Seto, 2012; for recent debates see Bailey, 2015; Cantor, 2015; Grundmann et al., 2017; Müller et al., 2014; Tozdan & Briken, 2017). That is not to say that prevention-focused approaches do not have this empirical assumption, but that it is secondary to the key aim of changing or preventing a particular behavior. The aim should therefore be to help service users to live healthy lives with their attractions (i.e., to gain self-acceptance), rather than subscribing to an assumption that all MAPs are likely to have compulsive thoughts or behaviors related to abusing children (Hocken, 2018). Such a focus on mental wellbeing is also consistent with empirically observed mental health deficits among MAPs, with higher-than-expected levels of loneliness (Elchuk et al., 2022; Jahnke et al., 2015a, b, 2018) thought suppression (Lievesley et al., 2020), and lower-than-average levels of generalized mental wellbeing including suicidal ideation (Cohen et al., 2020; Konrad et al., 2017; Lievesley et al., 2020) being reported among MAPs. Addressing such mental wellbeing issues has been suggested as a potential indirect route to sexual abuse prevention (Lievesley & Harper, 2021; Lievesley et al., 2020), given the associations between mental health issues, emotion regulation, and personality constructs with sexual offending among those with prior offense histories (Finkelhor, 1984; Gannon et al., 2012; Marshall, 2010; Ward & Beech, 2006; Ward & Siegert, 2002; Wielinga et al., 2021).

### Barriers to Help-Seeking and Effective Treatment Access

Despite the generally high numbers of MAPs suggesting a need for further support in relation to their sexual attractions (Elchuk et al., 2022; Levenson et al., 2017; Lievesley et al., 2020), there is also a frequently reported reluctance to engage in help-seeking due to the likelihood of experiencing stigmatization (Grady et al., 2019; Levenson & Grady, 2019b). MAPs often feel internalized stigma based on society’s messaging about pedophilia and thus can avoid seeking help due to the shame and fear of being reported to authorities based only on their reported attractions (Grady et al., 2019; Levenson & Grady, 2019b). These barriers are also recognized by professionals who are employed specifically to work with MAPs in preventative settings (Goodier & Lievesley, 2018; Parr & Pearson, 2019).

Skepticism about professional views and behaviors in relation to MAPs may not be unfounded. Across multiple studies of social work students and mental health professionals, we see a willingness to report MAPs to legal authorities due to explicit stigma and insecurities about professionals’ levels of competence, alongside a reluctance among some professionals to work with MAPs therapeutically at all (Beggs Christofferson, 2019; Jahnke et al., 2015a, b; Levenson & Grady, 2019a; Parr & Pearson, 2019; Stephens et al., 2021; Walker et al., 2022). Of course, within this context it is important to consider the psychological confusion for professionals in relation to reporting standards, and when disclosures need to be made from a legal or ethical perspective. That is, although some professionals are mandated reporters, this does not mean that disclosures of sexual attractions to children (in isolation) warrant the breaking of confidentiality (Walker et al., 2022). Instead, it is expected (and, in some cases, a legal requirement) that professionals will report patients and service users where there is evidence of offending, or concrete evidence of an imminent danger to an identifiable child. Nonetheless, professionals do report a willingness to report patients outside of these narrow parameters, including in response to an isolated disclosure of sexual attractions to children, or access to children in combination with such attractions (Stephens et al., 2021; Walker et al., 2022).

MAPs negative treatment encounters lead many to seek support in informal networks, such as via trusted friends and family, or online communities (Jones et al., 2021; Stevens & Wood, 2019). However, this leaves a gap in professional healthcare service provision, whereby genuine mental health issues remain untreated. As such, there have been calls to improve staff training and to reduce clinician stigma in healthcare settings to foster therapeutic environments that are accessible and responsive to MAPs’ needs (Goodier & Lievesley, 2018; Lievesley & Harper, 2021; McPhail et al., 2018; Parr & Pearson, 2019; Stephens et al., 2021).

### The Present Study: Primary Healthcare Providers’ Experiences, Beliefs, and Willingness to Work with MAPs

Due to convenience and accessibility, non-specialist health professionals such as general practitioners or family physicians are often the first to be accessed by MAPs when they do seek professional support (Levenson et al., 2020), but encounter this population incidentally as part of a broader job role. However, for the reasons identified, their experiences can be negative due to a combination of perceived clinician stigma, potentially incongruent treatment targets, and a lack of professional experience of working with individuals with sexual attractions to children. Specialists (operationalized here as healthcare professionals who specifically work with MAPs in therapeutic or preventative services) providing support in the management of sexual attractions to children receive advanced training to help MAPs to separate their attractions from their behavior and to live happy, healthy, and offense-free lives (Goodier & Lievesley, 2018; Jahnke, 2018; Parr & Pearson, 2019). Research exploring the role of specialists in MAPs’ help-seeking has identified how such services are often inaccessible or poorly publicized (Goodier & Lievesley, 2018; Parr & Pearson, 2019), which also leads to lower levels of service take-up.

Owing to this sparse accessibility of specialist treatment, it is important to explore the knowledge, comfort, and competence of healthcare providers working in general medical and mental health settings when faced with a minor-attracted presentation. Thus, this study explored the views of non-specialist healthcare professionals in managing MAP disclosures of their sexual attractions. We started this research program with no specific hypotheses owing to the almost total lack of background research that involves primary healthcare professionals. During data collection, one paper found how reporting decisions among Canadian mental health professionals were driven by stigma, the patient’s use of child sexual exploitation material, or their having access to children (Stephens et al., 2021). These findings, had they been available at the outset of this work, might have motivated us to form some tentative hypotheses, but they do serve to highlight the relevance of the constructs that we focus on in this work.

Primary healthcare providers fall between the general public (due to their lack of specific professional training) and specialist prevention-focused professionals (due to their experience in assisting patients with often complex and competing medical needs). As such, it was difficult to predict how constructs that we know are important to the experiences of MAPs would look in this sample. Instead, we started this project by developing a list of constructs that appear to be relevant to professional practice. These include experience with MAP disclosures, professional feelings of competence and comfort, clinician stigma, knowledge about sexual attractions to children, and anticipated treatment priorities. This choice of variables was motivated by the literature on MAPs’ concerns about seeking help in previous work (B4U-ACT, 2011; Grady et al., 2019; Levenson & Grady, 2019b), and the beliefs and experiences of those involved in specialist service provision (Goodier & Lievesley, 2018; Levenson & Grady, 2019a). Ultimately, our principal aim in this work was to provide a snapshot of current beliefs, understanding, and practice among primary healthcare professionals, and to identify opportunities for professional training and raise awareness of gaps in service provision for MAPs seeking professional support in these settings. As such, this paper presents a series of analyses exploring attitudinal differences between those with experiences of disclosure to those with no such patient history, and we explore the extent to which willingness to work with MAPs (and preferred treatment priorities) are predicted by constructs such as experience, knowledge, and clinician stigma.

## Method

### Participants

Recruitment for this research took several forms but focused on the targeted advertisement of the project to healthcare professionals. We made contact with national and regional professional bodies for general practitioners/family doctors, nurses, and psychiatrists in the UK, USA, Canada, Australia, and New Zealand (*N* = 21 organizations), requesting that the survey link was shared with their members. Although these invitations were generally well received,^1^ we did not receive a particularly high level of engagement through the survey link distributed by such bodies to their members. As such, we therefore supplemented this recruitment strategy with direct invitations to medical and mental health professionals on the social networking website LinkedIn (using targeted messaging on the basis of professional group memberships, as well as stated occupations in biographies) and direct emailing to GP practices and psychological professionals using publicly available distribution lists. Participant recruitment took place between April 2019 and February 2021, with a break imposed due to the global COVID-19 pandemic. That is, we ceased recruiting healthcare professionals during the height of the pandemic owing to the already numerous competing demands on their time. From the outset we aimed to recruit a minimum of 200 participants to ensure adequate sample sizes, in line with acceptable analytic rules-of-thumb and sample calculations based on our potential analyses. For example, conducting a factor analysis requires between 10- and 20-times the number of variables within a draft measure (Mundfrom et al., 2005) and our treatment priorities measure (see below) contained 11 items. In regression analyses it is common to target either 104 + *m* participants (where *m* equals the number of predictors in a model; Green, 1991), or 30 participants per predictor (VanVoorhis & Morgan, 2007). For exploring judgments of our progressive vignette (see below), an a priori sample calculation (assuming a medium effect size of *f* = 0.25, power = 95%, *α* = 0.05) suggested that we would need a minimum of 142 participants to detect a within-subjects effect.

A total of 277 people clicked on the survey link to read the information sheet, though there were no complete data-points for 31 of these. A further 26 participants declared their profession as administration-based and were ineligible to take part due to the non-therapeutic nature of their job role. This left a total of 220 participants (175 female, 45 male; *M*_age_ = 44.11 years, *SD* = 11.95) with usable data in the sample (i.e., they responded to questions in at least one of our planned analyses). We report exact sub-sample sizes throughout the paper, with lower numbers suggesting participant withdrawal prior to reaching particular questions. Specific withdrawal procedures required participants to contact the research team if their stopping of the survey indicated full withdrawal. This was made clear to participants at the time of obtaining informed consent. Nobody contacted us to withdraw from the study after starting the survey. Participants had been professionally qualified for an average of 15.06 years (*SD* = 11.16). We classified the occupational grouping of our participants (where provided) into “primary medical care” (*n* = 108), and “primary mental health care” (*n* = 103). Our decision to divide the sample into these two groups was driven by an understanding that general family doctors and physicians are usually the first point of contact with healthcare services, while also acknowledging that MAPs also report mental health issues as their key treatment targets (B4U-ACT, 2011). Over 90% of the sample was based in the UK. A full breakdown of participant characteristics is provided in Table 1.

**Table 1:** Sample demographics

Variable	*N*/% or *M*(*SD*)
*Sex*	
Female Male	175 (19.5%) 45 (20.5%)
Age (in years)	44.11 ± 11.95
Length of time qualified (in years)	15.06 ± 11.16
*Primary medical care (identifiable roles)*	
General practitioner/physician Nurse/healthcare assistant	71 (67.6%) 34 (32.4%)
*Primary mental health care (identifiable roles)*	
Psychologist Psychiatrist Counsellor/psychotherapist Mental health/wellbeing practitioner	76 (76.0%) 3 (3.0%) 14 (14.0%) 7 (7.0%)
*Country*	
UK USA Canada Australia New Zealand Other	201 (91.8%) 12 (5.5%) 0 (-) 1 (0.5%) 4 (1.8%) 1 (0.5%)
*Geographical context*	
Urban Rural	157 (72.0%) 61 (28.0%)

Percentages represent proportion of the total sample who provided responses to these questions

### Measures

#### Demographics

Participants were asked to provide details of their sex, age, length of qualification (which we used as an indicator of professional experience), location, and job role. These data are summarized in Table 1.

#### Experience and Anticipated Comfort

We asked participants to declare whether they had ever had a patient disclose a sexual attraction to children in the course of their professional practice. Those who answered yes were asked how many patients had disclosed such attractions, what these patients were seeking support for, how competent they were when working with these patients, and whether they had ever reported a patient to another agency. Those with no experience of such patient disclosures were asked about their hypothetical level of competence, and whether they would report a patient to another agency on the basis of their sexual attractions to children. We subsequently asked all participants a standardized list of questions about their feelings about working with patients who have a sexual attraction to children. These questions (with completed response numbers) were:

1. I would feel comfortable dealing with patients with this sexual interest (*n* = 213)
2. I would need support to deal with patients with this sexual interest (*n* = 213)
3. I would benefit from more training in how to deal with patients with this sexual interest (*n* = 213)
4. I would be willing to work/treat patients with this sexual interest (*n* = 198)
5. I would personally be able to treat patients with this sexual interest (*n* = 213)
6. I would want to refer patients with this sexual interest to appropriate services (*n* = 210)
7. I have appropriate services to refer patients with this sexual interest to (*n* = 211)

In each of these cases, responses were rated using a six-point scale anchored from “strongly disagree” to “strongly agree”, save for the initial question about experience with disclosures which was a binary choice (“yes” or “no”).

#### Knowledge About Pedophilia

To measure knowledge about pedophilia (as the most empirically studied chronophilia), we wrote a list of nine statements for the purposes of this research. Five of these were false, and four were true (for the full list of items, please see Supplementary Material for this article). We asked participants to select all statements that they believed were true, before recoding each statement to be scored 0 = incorrect and 1 = correct. Following this, we computed a “proportion correct” score for each participant (labeled “knowledge accuracy”) as an index of factual knowledge bout pedophilia (with a scoring range from 0 to 1; Kuder–Richardson-20 coefficient = 0.65). A total of 165 participants completed this measure. Descriptive and between-groups inferential statistics can be found in Supplementary Material.

#### Risk and Stigma

We measured general levels of perceived risk about sexual attractions to children using a self-created four-item measure to examine concerns about the potential risks posed by patients with sexual attractions to children. This measure asked participants to rate how likely they would be to suggest that such patients were a risk to the general public, and how likely they would be to report such patients to their manager, local safeguarding team (i.e., staff who are specifically employed to investigate issues related to abuse or neglect), and the police (as separate items). Each item was rated using a 6-point scale anchored from “strongly disagree” to “strongly agree” (scored 1–6), with an average score computed (*α* = 0.81). Higher scores were indicative of higher levels of concern over patients’ risk levels. A total of 175 participants completed this measure.

The stigmatization of people with sexual attractions to children was measured using Imhoff’s (2015) Stigma and Punitive Attitudes Scale (SPS). This is a questionnaire comprised of 35 items divided into four stigma domains:

1. Dangerousness (e.g., “Pedophiles are perverse sex offenders”; *α* = 0.76)
2. Intentionality (e.g., “If someone is pedophilic, there is nothing they can do about it”; *α* = 0.76)
3. Deviance (e.g., “Pedophiles are sick”; *α* = 0.71)
4. Punitive attitudes (“Pedophiles should be forced to undergo therapy”; *α* = 0.89)

Each item was framed using “pedophile” as the reference group, consistent with Imhoff’s (2015) original work. Participants responded to each statement using a 7-point scale anchored from 1 (strongly disagree) to 7 (strongly agree). Average scores were computed for each stigma domain, with higher scores indicating greater levels of stigmatization or punitive attitudes. A total of 154 participants completed this scale.

#### Treatment Priorities

We used the treatment priorities measure reported in B4U-ACT’s (2011) survey of MAPs in relation to their perceived treatment needs. However, we re-framed the items to be suitable for use with healthcare professionals. This measure consists of 11 items stating possible treatment targets (e.g., “To improve the patient’s self-concept,” “To help the patient control their sexual feelings,” and “To help the patient to extinguish or reduce an attraction to children”). Each statement was scored using a 10-point scale anchored from 1 (not a priority) to 10 (definitely a priority). Owing to the lack of use of this measure in previous peer-reviewed work, we present a factor analysis of the measure and report on its dimensionality and internal consistency within Results section. A total of 183 participants completed this measure.

#### Case Judgments

We self-produced a progressive vignette to explore how the disclosure of new case information might affect participant judgments and decision-making. The vignette was comprised of three parts, after each of which participants completed the same set of judgment questions. The exact wording of the vignette is provided below:

[Part 1—Attraction].

As a key professional in your service, you offer treatment and support to adults in the surrounding area. Luke schedules an appointment with you to discuss an ongoing issue relating to his sexual interests. During his appointment, Luke tells you he is experiencing sexual thoughts and fantasies that involve young children. More recently Luke has found these thoughts difficult to manage. He states that he has no interest in offending and has no criminal record. You are currently the only person available to deal with this.

[Part 2—Masturbation].

As you explore these issues with Luke, he discloses that he masturbates to these sexual fantasies.

[Part 3—Occupation].

In the same session, it emerges that Luke works as a school teacher.

At the end of each part of the vignette, participants responded to the following questions in a standardized order:

1. I would feel competent dealing with this patient.
2. I would feel comfortable dealing with this patient.
3. I would need support to deal with this patient.
4. How much of a sexual risk to children do you think Luke poses?
5. I would report this to my manager or supervisor.
6. I would report this to my local safeguarding team.
7. I would report this to my local police force.

Each statement was rated using a 6-point scale anchored from 1 (strongly disagree) to 6 (strongly agree), with the exception of question four where the scale was anchored from “very low risk” to “very high risk.” A total of 154 participants completed these vignette questions.

#### Unreported Measure

We adapted Feldman and Crandall’s (2007) 17-item measure of attributions about mental health to tap into the kinds of attributions that participants made about the nature of “sexual interests in children.” Each item (e.g., “To what extent are the clinical symptoms experienced by people with sexual interests in children completely sexual in nature?”) was rated using a seven-point slider, with each item using semantically appropriate anchors for the extreme values of 1 and 7. We explored the dimensionality of this measure in an earlier iteration of this paper. No meaningful factors underpinned the data, and as such we presented between-groups comparisons of each item. However, a reviewer of this earlier version suggested removing this owing to the degree of overlap between the attribution scale’s items and those contained within the SPS (Imhoff, 2015). As such, these analyses are not reported within the main body of this paper. In the interest of transparency, though, we present them within Supplementary Material accompanying this paper.

### Procedure

Participants were recruited via the targeted study advertisements described. Interested individuals were able to click the survey link to be taken to a screening page whereby they were asked whether they specifically work with people who have sexual interests in children (whether this be in a criminal justice role or in the community). Those who answered no were taken to a landing page that provided an overview of the study and its contents (participants who answered yes were screened out of this survey). We then collected affirmative consent before participants provided their demographic and occupational information. We then asked all participants about their experiences of having patients disclose sexual attractions to children, their perceived level of competence, reporting preferences, and anticipated future comfort. This section also included the measures of the perceived sexual offense risks posed by MAPs and participants’ treatment priorities when working with this population. The rest of the survey materials (related to stigma/attributions made about MAPs, hypothetical case judgments, and knowledge about MAPs) were randomized at the end of the survey. Upon completion of the survey, all participants were comprehensively debriefed and invited to contact the research team if they had any further questions.

## Results

Owing to the descriptive nature of this paper, we present frequency data below in relation to each of our measured variables. We supplement these data with some inferential testing (e.g., regression analyses directed at predicting reporting decisions and comfort, and comparative tests of judgments based on case information). These analyses are not tied to any pre-registered or a priori hypotheses and should therefore be read as exploratory.

### Past Experience and Anticipated Future Practice

We began with some basic descriptive analyses to establish the rate of which disclosures of sexual attractions to children had been made to our participants, and to estimate an average number disclosure experiences among those who had encountered patients with such sexual attractions. A total of 77 participants (35%) reported having had a patient disclose a sexual attraction to children in the course of their professional practice. On average, these participants had heard this disclosure from 9.86 patients (*SD* = 20.22), with a median figure of 2.00. However, a visual inspection of the boxplot of this variable suggested that the mean figure was skewed by 11 outliers who reported having been disclosed to by 20 (*n* = 2), 22 (*n* = 1), 30 (*n* = 3), 40 (*n* = 2), 80 (*n* = 1), and 100 patients (*n* = 2), respectively. Examining the qualitative responses to probes about their professional background, it appears that these participants have a history of working in secure mental health or forensic services, though none reported working specifically with individuals who have sexual convictions. Excluding these outliers led to an average number of disclosing patients of 2.90 (*SD* = 2.72), which may be closer to the true average number for primary healthcare professionals who have experience with this population (after removing these outliers, the median value was still 2.00). A Chi-square test demonstrated that patient disclosures were statistically over-represented among those working in primary mental health contexts (expected *n* = 36, observed *n* = 55) and statistically under-represented among those working in primary medical settings (expected *n* = 38, observed *n* = 18), *χ*^2^(2) = 31.63, *p* < 0.001, *φ* = 0.38. To clarify, “expected” *n*s are statistically derived and indicate the number of cases that we would have observed in each group if 35% prior experience rate had been present in each subsample.

We decided to compare the scores of those who both have and have not previously had patients disclose sexual attractions to children in order to compare anticipated experiences with MAPs to actual experiences. That is, if we were to find that those who had experienced such disclosures had more positive experiences than those who were merely anticipation working with MAP disclosures, this information could be used to allay anticipatory concerns when training primary healthcare professionals to work with this population. As shown in Table 2, those with experience of having patients disclose sexual attractions to children were more likely to feel competent in working with this group than those with no previous patient disclosures. Those with experience were also less likely to express a willingness to report such patients to another agency. When considering future anticipated practice, those with experience of having patients disclose a sexual attraction to children were significantly more likely to say that they would be comfortable and willing to work with this population, as well as being more confident in their ability to work with them. There were smaller differences in terms of the two groups’ self-perceived need for more training on how to work with MAPs, and desire to refer patients on to an appropriate service (where those with no experience were more likely to want more training, and to refer out of their service), though they did not differ in their doubt that such services currently exist.

**Table 2:** Professional opinions about working with MAPs, by experience of having had a patient disclose a sexual attraction to children

	Past experience?		
Statement	Yes	No	Inferential statistics
I felt/would feel competent dealing with patients with this sexual interest^a^	3.55 (1.55)	2.64 (1.36)	*t*(209) = 4.42, *p* < .001, *d* = 0.63
Have you ever reported/Would you report a patient with sexual interests in children to another agency?^a, b^	26 (exp = 46)	103 (exp = 83)	*χ*^2^(1) = 36.25, *p* < .001, *φ* = 0.42
I would feel comfortable dealing with patients with this sexual interest	3.76 (1.54)	2.80 (1.31)	*t*(211) = 4.78, *p* < .001, *d* = 0.67
I would need support to deal with patients with this sexual interest	4.73 (1.16)	5.30 (0.92)	*t*(211) = -3.93, *p* < .001, *d* = -0.53
I would benefit from more training in how to deal with patients with this sexual interest	4.93 (1.16)	5.27 (0.95)	*t*(211) = -2.30, *p* = .023, *d* = -0.32
I would be willing to work/treat patients with this sexual interest	4.56 (1.20)	4.10 (1.37)	*t*(196) = 2.37, *p* = .019, *d* = 0.36
I would personally be able to treat patients with this sexual interest	3.76 (1.45)	3.03 (1.48)	*t*(211) = 3.44, *p* = .001, *d* = 0.50
I would want to refer patients with this sexual interest to appropriate services	5.14 (0.90)	5.42 (0.89)	*t*(208) = -2.16, *p* = .032, *d* = 0.31
I have appropriate services to refer patients with this sexual interest to	2.74 (1.40)	2.40 (1.33)	*t*(209) = 1.75, *p* = .082, *d* = 0.25

Scores represent mean values, with a range from 1 to 6 with higher scores indicating a higher level of agreement. Standard deviations are presented in parentheses. Effect sizes show how those with experience of patient disclosures scored in comparison to those with no experience

^a^Wording differed based on past experience of working with disclosures. Those with no experience responded to the hypothetical framing

^b^Reporting question data refers to the observed number (vs. expected number) of people stating “yes”

### Stigma and Knowledge about Pedophilia

To explore the presence of stigmatized attitudes toward MAPs among healthcare professionals, we compared SPS scores within the current sample to those obtained from a large-scale public investigation whereby data were made available (Harper et al., 2021). This analysis was conducted to estimate the levels of stigma among professional groups. That is, previous work has highlighted that anticipated stigma is a barrier to MAPs seeking professional support (Grady et al., 2019; Levenson & Grady, 2019b), but there has been scant empirical attention paid to the actual levels of stigmatization among professional groups; nor has this stigma been broken down into its component domains of dangerousness, choice, and deviance attributions, alongside punitive attitudes. There has been a substantial amount of research published into public stigmatization of MAPs (e.g., Harper et al., 2018, 2021; Jahnke et al., 2015a, b) with high levels of stigma being reported. As such, we selected the public as a baseline level of stigma to compare the scores of professionals against, as MAPs are likely using societal stigma as a basis for their concerns.

We sampled a random selection of 110 participants from the public Harper et al. (2021) dataset to ensure approximately equivalent sample samples across each cell of our analysis before conducting a series of one-way analyses of variance (ANOVAs) to explore between-groups differences on each stigma domain. For reference, group-level statistics are presented in Table 3.

**Table 3:** Stigma domain scores, by group

	Group			
Stigma domain	General medical	Mental health	Public	Inferential statistics
Dangerousness	4.94 (0.93)	4.37 (1.08)	5.44 (0.93)	*F*(2, 261) = 26.29, *p* < .001, *η*^2^_*p*_ = 0.18
Intentionality	3.68 (0.95)	3.10 (0.78)	3.97 (1.50)	*F*(2, 261) = 18.64, *p* < .001, *η*^2^_*p*_ = 0.09
Deviance	4.92 (0.95)	4.28 (0.96)	5.15 (1.02)	*F*(2, 260) = 17.20, *p* < .001, *η*^2^_*p*_ = 0.12
Punitive attitudes	3.53 (1.03)	3.02 (0.77)	4.61 (1.28)	*F*(2, 258) = 53.36, *p* < .001, *η*^2^_*p*_ = 0.29

Scores represent mean values, with a range from 1 to 7 with standard deviations presented in parentheses. Higher scores indicate more stigmatized attitudes

We found a significant main effect of group on perceptions of dangerousness, where general medical professionals had significantly higher perceptions of dangerousness than mental health professionals (*M*_diff_ = 0.56, 95% CI_diff_ [0.19, 0.94], *p* = 0.001, *d* = 0.56) but lower perceptions of dangerousness than general public members (*M*_diff_ = −0.50, 95% CI_diff_ [−0.85, −0.15], *p* = 0.002, *d* = −0.54). Further, mental health professionals viewed MAPs as less dangerous than the general public (*M*_diff_ = −1.07, 95% CI_diff_ [−1.41, −0.72], *p* < 0.001, *d* = −1.07).

We found a significant main effect of group on perceptions of intentionality (i.e., choice over pedophilic sexual attractions), where general medical professionals had significantly higher perceptions of attraction choice than mental health professionals (*M*_diff_ = 0.60, 95% CI_diff_ [0.14, 1.06], *p* = 0.006, *d* = 0.68), but did not differ in these judgments to the public sample (*M*_diff_ = −0.30, 95% CI_diff_ [−0.72, 0.13], *p* = 0.272, *d* = −0.24). Mental health professionals perceived significantly less choice over these attractions than the public sample (*M*_diff_ = −0.90, 95% CI_diff_ [−1.32, −0.48], *p* < 0.001, *d* = −0.75).

We found a significant main effect of group on perceptions of MAP deviance (i.e., mental ill health). General medical professionals had significantly higher perceptions of deviance than mental health professionals (*M*_diff_ = 0.64, 95% CI_diff_ [0.25, 1.02], *p* < 0.001, *d* = 0.66), but did not differ in these judgments to the public sample (*M*_diff_ = −0.22, 95% CI_diff_ [−0.58, 0.14], *p* = 0.414, *d* = −0.22). Mental health professionals scored significantly lower on this stigma domain than the public sample (*M*_diff_ = −0.86, 95% CI_diff_ [−1.21, −0.50], *p* < 0.001, *d* = −0.86).

We found a significant main effect of group on punitive attitudes toward MAPs. The general medical professionals in our sample were significantly more punitive than mental health professionals (*M*_diff_ = 0.51, 95% CI_diff_ [0.09, 0.93], *p* = 0.012, *d* = 0.56) but less punitive than the public members (*M*_diff_ = −1.08, 95% CI_diff_ [−1.47, −0.69], *p* < 0.001, *d* = −0.93). Mental health professionals were significantly less punitive than the public sample (*M*_diff_ = −1.59, 95% CI_diff_ [−1.98, −1.20], *p* < 0.001, *d* = −1.51).

### Treatment Priorities

Because the treatment priorities measure has not been used in peer-reviewed research before, we initially conducted an exploratory factor analysis to examine its dimensionality (see Supplementary Material). Three factors were retained, related to: (1) general mental health concerns, (2) the forensic control or change of sexual attractions to children, and (3) living with a stigmatized sexual attraction pattern.

**Fig. 1:**
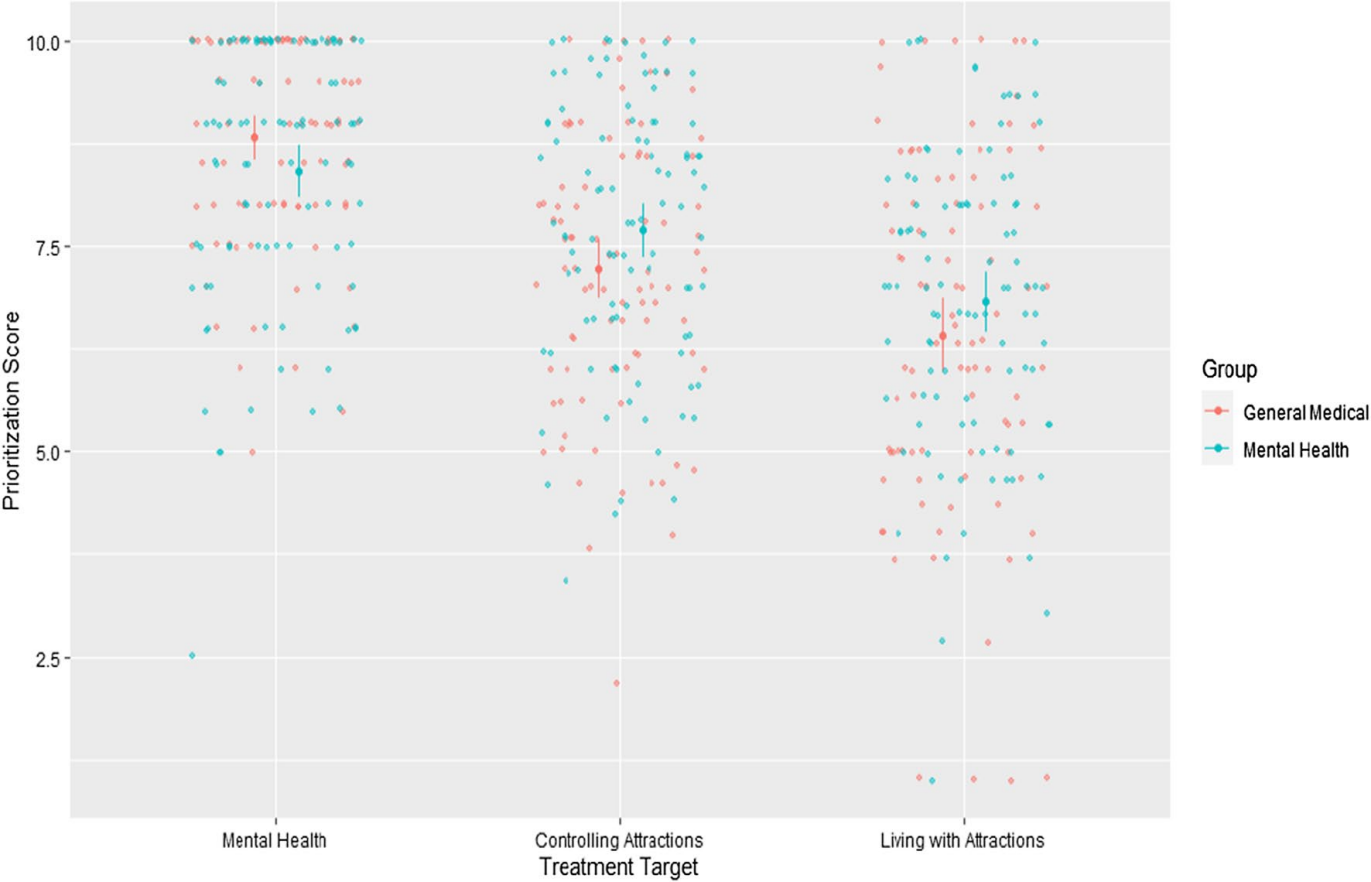
Treatment target prioritization, by professional group. Individual dots represent raw datapoints. Error bars represent the 95% CI of the mean. Descriptive statistics are provided in Supplementary Materials to preserve the readability of the plot

After calculating average scores for each treatment priority factor, we conducted a 2 (professional group: primary medical vs. primary mental health; between-participants) × 3 (treatment target; within-participants) analysis of variance (ANOVA) to compare the extent to which participants endorse each treatment priority cluster, and to explore whether this level of endorsement was consistent across professional groups. This was important after acknowledging how MAPs themselves appear to have primary treatment targets related to health and wellbeing, with a lower emphasis on controlling their attractions or changing them (B4U-ACT, 2011). In this analysis, we found a significant main effect of treatment target, *F*(2, 362) = 92.7, *p* < 0.001, *η*^2^_*p*_ = 0.34. Here, mental health targets were significantly more likely to be prioritized over both controlling sexual attractions (*M*_diff_ = 1.16, 95% CI_diff_ [0.84, 1.48], *p* < 0.001, *d* = 0.74) and living with stigmatized attractions (*M*_diff_ = 1.97, 95% CI_diff_ [1.64, 2.30], *p* < 0.001, *d* = 1.14). Further, targets related to controlling attractions were significantly higher in terms of treatment priority than those related to living with stigmatized attractions (*M*_diff_ = 0.81, 95% CI_diff_ [0.41, 1.21], *p* < 0.001, *d* = 0.45). There was also a statistically significant interaction between treatment target and grouping variable, *F*(2, 362) = 5.35, *p* = 0.005, *η*^2^_*p*_ = 0.03. This was attributable to marginally higher prioritization of mental health concerns and lower prioritization of targets for controlling attractions among primary medical professionals (e.g., general practitioners). However, these differences did not reach the arbitrary threshold for statistical significance within the univariate post hoc tests (Bonferroni-corrected *p*s = 0.056 and 0.053, respectively). Figure 1 demonstrates these trends.

We next entered professional group, past experience of patient disclosure, stigma scale scores, and knowledge accuracy as predictors in a linear regression predicting prioritization scores for each of the treatment targets. In running such a regression model, we hoped to identify the variables that are associated with treatment targets that are congruent with those also endorsed by MAPs. These variables might subsequently become targets for intervention through training programs, as having a degree of congruence between professional and patient goals is known to be associated with treatment engagement and success (Arnow & Steidtmann, 2014; Browne et al., 2019; Horvath et al., 2011). Correlational analyses demonstrated linearity between the measured variables, while skewness and kurtosis were all within acceptable limits (see Table 4). All regression coefficients are presented in Table 5.

**Table 4:** Zero-order correlations between treatment priorities, stigma scores, and knowledge about pedophilia

	1	2	3	4	5	6	7	8
1. Mental health priority	–							
2. Controlling attractions priority	.30^***^	–						
3. Living with attractions priority	.43^***^	.24^**^	–					
4. SPS Dangerousness scale	−.06	.21^**^	−.16^*^	–				
5. SPS Intentionality scale	−.23^**^	.05	−.23^**^	.46^***^	–			
6. SPS Deviance scale	.03	.25^**^	−.26^**^	.51^***^	.34^***^	–		
7. SPS Punitive Attitudes scale	−.12	.10	−.26^**^	.61^***^	.53^***^	.45^***^	–	
8. Knowledge accuracy	.09	−.06	.16^*^	−.47^***^	−.46^***^	−.28^**^	−.50^***^	–
*M* (SD)	8.61 (1.44)	7.47 (1.65)	6.63 (2.00)	4.66 (1.04)	3.37 (0.92)	4.61 (1.01)	3.27 (0.94)	0.69 (0.20)
Skewness	−1.06	−0.40	−0.46	−0.10	0.57	−0.48	1.26	−0.44
Kurtosis	1.00	−0.38	0.16	−0.20	1.12	0.10	2.02	0.36

Treatment priority scores ranged from 1–10, with higher scores indicating greater prioritization. SPS scores range from 1–7, with higher scores indicating greater stigmatization. Knowledge accuracy ranges from 0–1, with higher scores indicating greater accuracy

*p < .05 **p < .01 ***p < .001

**Table 5:** Predictors of treatment target prioritization

	Mental health concerns		Control of attractions		Living with stigma	
	*B*	*p*	*B*	*p*	*B*	*p*
*Constant*	10.27 [7.42, 13.13]	< .001	1.79 [−1.42, 5.00]	.273	9.02 [5.05, 12.99]	< .001
Professional group	−0.57 [−1.10, −0.03]	.038	1.02 [0.42, 1.62]	.001	−0.02 [−0.76, 0.72]	.960
Past experience	0.24 [−0.28, 0.77]	.361	0.42 [−0.18, 1.01]	.167	0.19 [−0.55, 0.92]	.613
SPS Dangerousness scale	0.03 [−0.28, 0.33]	.849	0.34 [−0.00, 0.68]	.052	0.20 [−0.22, 0.63]	.345
SPS Intentionality scale	−0.47 [−0.78, −0.16]	.003	0.01 [−0.34, 0.36]	.944	−0.26 [−0.70, 0.17]	.229
SPS Deviance scale	0.12 [−0.16, 0.39]	.399	0.44 [0.14, 0.75]	.005	−0.39 [−0.77, −0.01]	.042
SPS Punitive Attitudes scale	−0.10 [−0.44, 0.24]	.555	−0.12 [−0.50, 0.26]	.546	−0.34 [−0.81, 0.13]	.157
Knowledge accuracy	0.04 [−1.36, 1.44]	.954	0.29 [−1.29, 1.86]	.720	0.27 [−1.67, 2.22]	.782

Figures inside square brackets represent 95% CIs of the unstandardized *B* coefficient. 95% CIs that to not include “0” as a possible value indicates a significant predictor of treatment prioritization. “Professional group” was coded 1 = general medical, 2 = mental health

The model predicting mental health treatment priorities was statistically significant and explained 11.9% of the variance in treatment prioritization, *R*^2^ = 0.119, *F*(7, 140) = 2.69, *p* = 0.012. Professional group (specifically, being a primary medical professional) was associated with higher prioritization of mental health needs, while increased perceptions of intentionality (i.e., perceiving people with pedophilic sexual attractions as having a choice) were associated with lower prioritization of mental health treatment targets.

The model predicting treatment targets aimed at controlling or changing sexual attractions to children was statistically significant and explained 15.1% of the variance in treatment prioritization, *R*^2^ = 0.151, *F*(7, 140) = 3.56, *p* = 0.002. Professional group (specifically, being a mental health professional) was associated with a greater prioritization of control-related treatment targets. This treatment target was also prioritized by those who perceive people with pedophilic sexual attractions as being more deviant.

The model predicting treatment targets designed to address living with societally stigmatized sexual attractions was statistically significant and explained 10.8% of the variance in treatment prioritization, *R*^2^ = 0.108, *F*(7, 140) = 2.42, *p* = 0.023. Addressing these patient concerns about living with stigma was prioritized by those who do not perceive people with pedophilic sexual attractions as being particularly deviant.

In no model was either past experience of having patients disclose sexual attractions to children or knowledge accuracy about pedophilia significant predictors of treatment target prioritization.

### Willingness to Treat MAPs

To explore the factors related to willingness to treat MAPs in healthcare settings, we ran a linear regression predicting scores on the willingness to treat single-item variable. Within this model, we planned to enter professional group, prior experience of patient disclosures, perceived competence, anticipated comfort, desires for both more support and more training, each of the four stigma domains, risk concerns, knowledge about pedophilia, and all three treatment targets. Correlational analyses demonstrated broad linearity between the measured variables, but we did observe multicollinearity between perceived competence and anticipated comfort (*r* = 0.84, *p* < 0.001). To account for this, we removed perceived competence from the model for two reasons. First, the comfort item was framed as future-looking (rather than competence perceived during past disclosures for some of the sample). Second, we felt that including desires for more support and training could account for some of the variance explained by a perceived competence variable. Skewness and kurtosis were generally all within acceptable limits (see Table S8 in Supplementary Material). We used G*Power (Faul et al., 2007) to conduct a post hoc power analysis to ensure that this regression was not underpowered. Converting our observed *R*^2^ value (0.41) to Cohen’s *f*^2^ (0.70), we found that our analysis sample (i.e., those with full data on all variables, and thus eligible for inclusion) of 139 gave us 100% power to detect significant effects at *p* < 0.05 level, and 99.9% power to detect effects at the *p* < 0.001 level.

All regression coefficients are presented in Table 6. The model was statistically significant and explained 41% of the variance in participants’ willingness to treat MAPs, *R*^2^ = 0.410, *F*(14, 124) = 6.26, *p* < 0.001. Within the model, anticipated comfort and having mental health-related treatment targets were associated with a greater willingness to work with this population. In contrast, prioritizing the control of sexual attractions was associated with resistance to treating MAPs.

**Table 6:** Predictors of willingness to treat MAPs

	*B* (*SE*)	*t*	*p*	95% CI (*B*)
*Constant*	0.95 (1.55)	0.61	.544	[−2.13, 4.02]
Professional group	−0.14 (0.24)	−0.59	.558	[−0.61, 0.33]
Past experience	−0.23 (0.23)	−1.03	.306	[−0.68, 0.22]
Anticipated comfort	0.48 (0.08)	5.90	< .001	[0.32, 0.64]
Need for more support	−0.06 (0.11)	−0.50	.616	[−0.27, 0.16]
Desire for more training	0.17 (0.10)	1.65	.101	[−0.03, 0.37]
SPS Dangerousness scale	−0.09 (0.13)	−0.72	.475	[−0.34, 0.16]
SPS Intentionality scale	−0.15 (0.13)	−1.17	.245	[−0.41, 0.11]
SPS Deviance scale	0.19 (0.12)	1.61	.110	[−0.04, 0.43]
SPS Punitive Attitudes scale	0.01 (0.14)	0.07	.943	[−0.38, 0.30]
Risk concerns	0.09 (0.11)	0.81	.422	[−0.13. 0.31]
Knowledge accuracy	0.35 (0.57)	0.61	.545	[−0.78, 1.47]
Mental health priorities	0.25 (0.08)	3.12	.002	[0.09, 0.40]
Controlling attractions priorities	−0.18 (0.07)	−2.72	.007	[−0.32, −0.05]
Living with stigma priorities	0.07 (0.06)	1.20	.232	[−0.43, 0.18]

95% CIs that do not include “0” as a possible value indicates a significant predictor of willingness to treat MAPs. “Professional group” was coded 1 = general medical, 2 = mental health

### Hypothetical Case Judgments

**Fig. 2:**
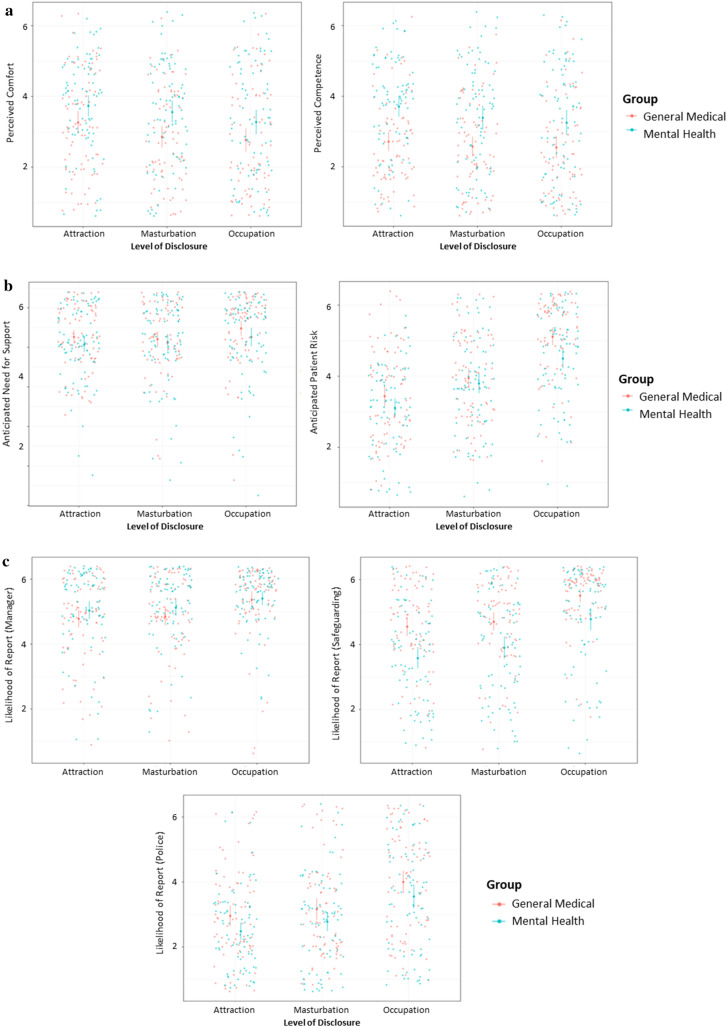
**a** Professional comfort and judgments, by level of disclosure. Individual dots represent raw datapoints. Error bars represent ± 1 SE of the mean. Descriptive statistics are provided in Supplementary Materials to preserve the readability of the plot. **b** Professional support and risk judgments, by level of disclosure. Individual dots represent raw datapoints. Error bars represent ± 1 SE of the mean. Descriptive statistics are provided in Supplementary Materials to preserve the readability of the plot. **c** Professional reporting intentions, by level of disclosure. Individual dots represent raw datapoints. Error bars represent ± 1 SE of the mean. Descriptive statistics are provided in Supplementary Materials to preserve the readability of the plot

We conducted a series of seven two-way mixed-design ANOVAs to explore the effects of the progressive vignette on participants’ judgments and hypothetical decision-making. An analysis of each judgment question was conducted separately. This analysis was designed to explore whether specific case details changed the beliefs or anticipated behaviors among participants. In each test, the within-subjects independent variable was the stage of the vignette, with three levels (attraction disclosure, masturbation disclosure, and occupation disclosure), while the between-subjects independent variable was professional group, with two levels (general medical and mental health professional). For clarity, average scores for each outcome at each stage of the vignette are presented in Table 7. Due to the lack of significant interactions between the two independent variables, whole-sample averages are displayed in the table, while graphical depictions of the trends for each outcome across the vignette are presented in Fig. 2a–c.

**Table 7:** Sample averages for judgment outcomes at each stage of the vignette

	Level of disclosure		
	Attraction	Masturbation	Occupation
Competence	3.23 (0.11) *M*_diff_ = −0.22, 95% CI_diff_ [−0.36, −0.07], *p* = .001, *d*_z_ = −0.10	3.02 (0.12) *M*_diff_ = −0.04, 95% CI_diff_ [−0.20, 0.11], *p* > .999, *d*_z_ = −0.02	2.98 (0.12)
Comfort	3.55 (0.11) *M*_diff_ = −0.29, 95% CI_diff_ [−0.45, −0.14], *p* < .001, *d*_z_ = −0.15	3.25 (0.11) *M*_diff_ = −0.15, 95% CI_diff_ [−0.30, −0.01], *p* = .037, *d*_z_ = −0.07	3.10 (0.12)
Need support	5.13 (0.07) *M*_diff_ = 0.01, 95% CI_diff_ [−0.12, 0.14], *p* > .999, *d*_z_ = 0.00	5.14 (0.08) *M*_diff_ = 0.21, 95% CI_diff_ [0.06, 0.36], *p* = .003, *d*_z_ = 0.15	5.34 (0.08)
Risk judgment	3.26 (0.10) *M*_diff_ = 0.57, 95% CI_diff_ [0.43, 0.72], *p* < .001, *d*_z_ = 0.32	3.83 (0.10) *M*_diff_ = 0.94, 95% CI_diff_ [0.76, 1.11], *p* < .001, *d*_z_ = 0.53	4.77 (0.10)
Report (supervisor)	4.85 (0.10) *M*_diff_ = 0.07, 95% CI_diff_ [−0.05, 0.19], *p* = .420, *d*_z_ = 0.04	4.93 (0.10) *M*_diff_ = 0.39, 95% CI_diff_ [0.25, 0.54], *p* < .001, *d*_z_ = 0.23	5.32 (0.08)
Report (safeguarding)	4.07 (0.12) *M*_diff_ = 0.22, 95% CI_diff_ [0.07, 0.36], *p* = .001, *d*_z_ = 0.10	4.28 (0.12) *M*_diff_ = 0.80, 95% CI_diff_ [0.62, 0.98], *p* < .001, *d*_z_ = 0.40	5.08 (0.11)
Report (police)	2.64 (0.10) *M*_diff_ = 0.27, 95% CI_diff_ [0.13, 0.41], *p* < .001, *d*_z_ = 0.14	2.91 (0.11) *M*_diff_ = 0.80, 95% CI_diff_ [0.61, 0.99], *p* < .001, *d*_z_ = 0.37	3.71 (0.13)

All outcomes rated using a 1–6 scale, with high scores indicating more competence, comfort, and desire for support, higher risk judgments, and a greater willingness to report the patient to their supervisor, safeguarding team, or local police force. Data represent estimated marginal means with standard error presented in parentheses. Mean differences describe differences between each level of disclosure, rounded to two decimal places. Mean difference information on the left demonstrates differences between the “attraction” and “masturbation” levels, while information on the right demonstrates differences between the “masturbation” and “occupation” levels

There was a significant effect of disclosure level on judgments of competence in dealing with the hypothetical case, *F*(2, 304) = 13.34, *p* < 0.001, *η*^2^_*p*_ = 0.08. Within this model, there was a significant reduction in perceived competence when they were told about the patient’s masturbation to sexual thoughts involving children although hearing about the patient’s occupation did not significantly alter competence perceptions any further. There was also a significant effect of group on perceived competence, *F*(1, 152) = 13.83, *p* < 0.001, *η*^2^_*p*_ = 0.08. Here, mental health professionals (*M* = 3.43, *SE* = 0.15) scored higher on perceived competence than general medical professionals (*M* = 2.64, *SE* = 0.15), *M*_diff_ = 0.79, 95% CI_diff_ [0.36, 1.21], *p* < 0.001, *d* = 0.72. There was no interaction between the two independent variables, *F*(1, 304) = 13.83, *p* = 0.115, *η*^2^_*p*_ = 0.01.

There was a significant effect of disclosure level of comfort in relation to working with the hypothetical case, *F*(2, 304) = 23.67, *p* < 0.001, *η*^2^_*p*_ = 0.14. Within this model, there was a significant reduction in comfort when participants were told about the patient’s masturbation activity, and comfort reduced significantly further when finding out that he worked in a school. There was also a significant effect of group on perceived competence, *F*(1, 152) = 5.99, *p* = 0.016, *η*^2^_*p*_ = 0.04. Here, mental health professionals (*M* = 3.51, *SE* = 0.16) scored higher on anticipated comfort than general medical professionals (*M* = 2.97, *SE* = 0.16), *M*_diff_ = 0.54, 95% CI_diff_ [0.11, 0.98], *p* = 0.016, *d* = 0.54. There was no interaction between the two independent variables, *F*(1, 304) = 2.21, *p* = 0.112, *η*^2^_*p*_ = 0.08.

There was a significant effect of disclosure level on the extent to which participants felt that they would need further support in working with this patient, *F*(2, 304) = 7.20, *p* = 0.001, *η*^2^_*p*_ = 0.05. Within this model, there was no change in the need for support after hearing about the patient masturbating to sexual thoughts involving children, the need for support rose significantly upon learning about his occupation. There was no effect of group on the need for support, *F*(1, 152) = 1.45, *p* = 0.231, *η*^2^_*p*_ = 0.01. There was also no interaction between the independent variables, *F*(1, 304) = 0.61, *p* = 0.544, *η*^2^_*p*_ < 0.01. Unsurprisingly, these need for support trends mirrored those related to the likelihood of reporting the patient to the participant’s manager or supervisor, with a significant effect of disclosure level (*F*(2, 304) = 31.34, *p* < 0.001, *η*^2^_*p*_ = 0.17) but no effect of group (*F*(1, 152) = 1.33, *p* = 0.251, *η*^2^_*p*_ = 0.01), nor an interaction between these variables (*F*(2, 304) = 2.71, *p* = 0.068, *η*^2^_*p*_ = 0.02).

There was a significant effect of disclosure level on judgments of participants’ judgments of the patient’s risk of sexual offending, *F*(2, 304) = 212.82, *p* < 0.001, *η*^2^_*p*_ = 0.58. Within this model, there was a significant rise in perceived risk upon hearing about the patient’s engagement in masturbation habits, with risk perceptions rising significantly further when the patient’s job role was disclosed. There was no effect of group in relation to sexual risk judgments, *F*(1, 152) = 3.69, *p* = 0.057, *η*^2^_*p*_ = 0.02. There was, however, a significant interaction between the two variables, *F*(2, 304) = 5.20, *p* = 0.006, *η*^2^_*p*_ = 0.03. Here, the groups did not differ in their risk perceptions at either the “attraction” (*M*_diff_ = 0.28, 95% CI_diff_ [−0.12, 0.68], *p* = 0.170, *d* = 0.26) or “masturbation” (*M*_diff_ = 0.15, 95% CI [−0.25, 0.56], *p* = 0.457, *d* = 0.12) levels of disclosure. However, general medical professionals inferred significantly more risk (*M* = 5.09, *SE* = 0.14) than mental health professionals (*M* = 4.47, *SE* = 0.14) when the patient disclosed working as a teacher, *M*_diff_ = 0.62, 95% CI_diff_ [0.23, 1.01], *p* = 0.002, *d* = 0.51.

There was a significant effect of disclosure level on participant’s likelihood of reporting the patient to a safeguarding team, *F*(2, 304) = 108.66, *p* < 0.001, η^2^_p_ = 0.40, whereby reporting intentions significantly increased when it was disclosed that he was masturbating to sexual thoughts involving children, and again when the patient’s work context was revealed. There was also a significant effect of group on safeguarding reporting intentions, *F*(1, 152) = 17.00, *p* < 0.001, *η*^2^_*p*_ = 0.10. Here, general medical professionals (*M* = 4.91, *SE* = 0.15) were more likely to express a willingness to report the patient to safeguarding services than mental health professionals (*M* = 4.06, *SE* = 0.14), *M*_diff_ = 0.84, 95% CI_diff_ [0.44, 1.25], *p* < 0.001, *d* = 0.67.

There was a significant effect of disclosure level on participants’ likelihood of reporting the patient to their local police force, *F*(2, 324) = 103.26, *p* < 0.001, *η*^2^_*p*_ = 0.39, with reporting intentions increasing significantly upon hearing about the patient’s masturbation activity, and still further when participants learned that he worked in a school. There was no effect of group on police reporting intentions, *F*(1, 152) = 3.66, *p* = 0.057, *η*^2^_*p*_ = 0.02. Similarly, there was no interaction between the two independent variables, *F*(2, 304) = 0.31, *p* = 0.736, *η*^2^_*p*_ < 0.01.

## Discussion

Owing to the exploratory nature of this project, we now present a discussion of our findings in relation to three specific areas that appear to be important in the provision of services for MAPs.

### Professional Experiences of Client Disclosure

In this initial snapshot study of attitudes, beliefs, and willingness to work with MAPs, we have explored a range of different issues that highlight a need for greater levels of training and ongoing professional support for healthcare providers who may incidentally encounter patients with sexual attractions to children. Within our sample, 35% of participants had been disclosed to by a patient about their sexual attractions to children, with an average of around three patients disclosing per professional (after removing outliers from this calculation). Disclosures were statistically over-represented in mental health services compared to general medical contexts (e.g., to general practitioners or family physicians). This disparity may be explained by MAPs’ treatment needs (B4U-ACT, 2011; Elchuk et al., 2022; Jahnke et al., 2015a, b). That is, they may approach primary medical practitioners in the first instance with attraction-related mental health needs and then disclose the underlying reasons for their difficulties when working more closely with professionals whom they feel are more qualified or competent to help, or sympathetic to their position (Grady et al., 2019).

Experience of patient disclosures was associated with lower levels of perceived incompetence and anticipated discomfort in working with MAPs in the future, alongside lower perceptions of a need for more support or training in how to work with this group. Anticipated comfort in working with MAPs was predictive of an increased willingness to treat them in the future, even after controlling for other risk indices that were nonsignificantly associated with such a willingness. This may suggest that global feelings of unease or discomfort are more important to address than are specific domains of stigmatization, such as perceptions of dangerousness among MAPs or beliefs about choice or control over sexual attractions, in creating an environment within which MAPs are initially welcome. However, as will be explored, addressing these specific domains of stigma may also be important in maintaining effective therapeutic goals and relationships with patients who present with treatable medical or psychological needs.

Both groups (i.e., those with and without experiences of patient disclosures) scored relatively highly on the need for further training, highlighting a need for education to be offered to professionals involved in primary (i.e., non-specialist) healthcare provision, to empower them to feel comfortable and competent when making clinical decisions about MAPs under their care. Focusing on MAP-related issues in training programs for non-specialist medical and mental health professionals may not be considered a priority for some, with a minority of our sample (albeit sizeable at 35%) reporting ever having contact with patients disclosing sexual attractions to children. This training, however, might easily be incorporated into a broader module about working with atypical sexuality and sexual identity in a broader sense. Practitioners receive minimal training on working with patients presenting a host of sex-related issues (Clegg et al., 2016; Stott, 2013; Ucsnik et al., 2018), and we see education of sexual attractions to children being a part of this more general expansion of professional training.

Specifically in relation to the content of such education, training should focus on factual information about MAPs and the nature of sexual attractions to children in the first instance, as we observed a lack of basic knowledge about definitional issues related to such attractions within the current sample. That is, correct responses were provided to the knowledge measure just 65% (general medical professionals) or 74% (mental health professionals) of the time. In terms of identifying the fundamental definition of pedophilia (i.e., a sexual attraction to children below the age of 11 years; Seto, 2017), just 23% and 43%, respectively, responded correctly. Education around risk, choice over sexual attractions, and controllability would also be beneficial, particularly for general medical professionals. These participants viewed MAPs as more likely to be dangerous and less likely to be able to control their behavior (when compared to mental health professionals). In addition, there was a simultaneous view observed among general medical professionals that an attraction is an avoidable choice, and difficult to control from a behavioral perspective. This is potentially indicative of the view that MAPs are in some way inevitably going to offend because of their sexual attractions, which is consistent with the views held by many untrained members of the public (Lawrence & Willis, 2021). Professional training on what we currently know about the now recognized groups of individuals living with sexual attraction to children who are non-offending (i.e., living with and controlling their sexual attractions; Cantor & McPhail, 2016) would help to challenge these blanket views, as would exploration of the emerging understanding that primary sexual attractions to children are largely unchangeable (Grundmann et al., 2016; Seto, 2012).

### Identifying Appropriate Treatment Goals

In time, educational initiatives with healthcare providers might explore more nuanced issues related to working with MAPs, including issues related to the development of sexual attractions to children and the selection of appropriate treatment options. In general, our sample rated mental health-related concerns as a treatment priority above targets related to controlling sexual attractions (including changing attraction patterns) or coping with social stigmatization. This is broadly consistent with how MAPs conceptualize their treatment needs in surveys conducted within the MAP community, with MAPs reporting social stigmatization as being a driver of poorer mental health in a range past work (B4U-ACT, 2011; Grady et al., 2019; Jahnke et al., 2015a, b, 2018; Levenson & Grady, 2019b; Levenson et al., 2017).

As mentioned previously, addressing misconceptions and challenging stigma about people who have sexual attractions to children are important issues to tackle as professionals seek to work collaboratively and constructively with MAPs. We know from the general healthcare literature that perceptions of professionals’ genuine care, and collaborative approaches to setting treatment goals and approaches can improve the therapeutic alliance between healthcare professionals and their patients (Elvins & Green, 2008; Locati et al., 2019; Nienhuis et al., 2018), with this improving both treatment engagement and efficacy (Arnow & Steidtmann, 2014; Browne et al., 2019; Horvath et al., 2011). Assisting professionals to make good decisions about treatment goals (i.e., addressing the healthcare needs that their patients present with, rather than their inferred needs stemming from stigmatizing assumptions of risk; see below) is likely to be a positive first step in professional education.

Although mental health issues were prioritized by participants, the average treatment prioritization scores related to controlling or changing attractions were relatively high within the current dataset, with a mean score of 7.42 on a 1–10 scale. This may in some way be tied with relatively high levels of belief in the intentionality (i.e., choice) over sexual attractions to children, especially among general medical professionals who did not differ in this regard to non-professional members of the public. This is an important observation, as such beliefs were associated with a lessened focus on MAPs’ mental health treatment priorities, which in turn was associated with a lower level of willingness to work with this population. Similarly, general medical professionals held similar levels of belief about the psychological deviance of MAPs to members of the public with no medical training, with this predicting a greater level of prioritization of treatment targets related to controlling or changing sexual attractions. Endorsing these treatment targets was in turn associated with a lower level of willingness to work with MAPs.

As such, addressing stigmatization across these domains specifically might be a potential future step when designing professional training packages for healthcare professionals. One such successful example of a training approach incorporates indirect contact with MAPs, where Jahnke et al. (2015a), b) presented a documentary film to therapists that contained first-hand accounts from MAPs about their experiences of having sexual attractions to children. In doing so, stigma levels were reduced over an average follow-up period of 2–3 weeks. Psychologically, the process of narrative humanization is activated in this intervention (Harper et al., 2018) whereby widely held social stereotypes begin to be broken by the experience of seeing MAPs as people, rather than their label. As such, traditional education practices (e.g., considerations of treatment philosophies and critical engagement with the mental health and abuse prevention literatures) might be supplemented with exposure to MAPs (either directly within the training room or indirectly via documentary films). Some researchers using this approach to stigma reduction have speculated about whether such educational approaches contribute to changes in behavior toward MAPs (Harper et al., 2021) and have advocated for studies to incorporate more measures of behavioral change (e.g., support for MAP wellbeing schemes, contact with MAPs) as an outcome in future research. We see clinician behavior as another potential outcome in this search for an answer about whether changes in self-reported stigma also contribute to changes in real-world behavior toward MAPs.

All participants appeared to agree that there is a lack of available services for them to refer MAPs on to if this is either desired or required. Although it is true that prevention initiatives are still in their infancy, there is a growing global effort to develop primary and secondary prevention strategies, with support services on the incline (for an overview, see Christiansen & Martinez-Dettamantim, 2018). This finding may therefore highlight an issue with awareness of services. However, we do also know that the infancy of these initiatives means that services are often not accessible or available for reasons of geography, price, or service capacity (Shields et al., 2020). Emerging research using retrospective accounts of individuals convicted of sexual offenses also highlight how many are turned away or dismissed for treatment by health professionals (Levenson et al., 2017; Lievesley et al., 2016). The lack of formal services is also reflected in the growing body of online support groups set up by MAPs (Malone, 2016). The reasons for this are multitude, but are commonly cited to revolve around lack of knowledge and stigmatizing attitudes on the behalf of professionals (Grady et al., 2019; Levenson et al., 2017; Levenson & Grady, 2019b; Shields et al., 2020). Services should look to increase specialist training so that professionals are able to respond with compassion to MAPs and aims and outcomes of any preventative initiative should be designed around the health needs of MAPs as opposed to being driven by stigmatizing and often inaccurate views around this sexual attraction (B4U-ACT, 2017; Lievesley et al., 2018).

### Professional Decision-Making and Reporting to Authorities

Looking at specific case details and how they affect decision-making, we found that learning of a MAPs’ masturbation to sexual thoughts involving children reduced perceived competence and comfort in working with him, increased perceptions of his risk of sexual offending, and a greater level of intention to report him to a local safeguarding team or police force. These trends were exacerbated still further when it was disclosed that he worked in a school, with comfort further reducing, desires for support increasing, and raised perceptions of risk and intentions to report to supervisors, local safeguarding teams, and the police. Although intentions to report to the police were lower than intentions to report to clinical supervisors or local safeguarding teams, learning about the patient’s occupation led to an increase in police reporting intentions that placed the mean value above the scale’s mid-point, indicating a general willingness to report the patient to law enforcement officials even in the absence of any evidence of offending behavior. This may be tied up with the inherently subjective standard of determining “meaningful risk” in cases whereby sexual abuse may be suspected. That is, the vignette’s final level, wherein the patient’s occupation was disclosed, may have hit the threshold for our participants to perceive a “meaningful risk” due to the patient’s access to children. However, as noted by Walker et al. (2022):

under duty to warn regulations, without identifying a potential victim or indicating a specific, upcoming threat, there is not sufficient justification for reporting. Relatedly, under mandated reporting guidelines, providers are required to break confidentiality with their clients only when they have a reasonable suspicion that a specific child has been subjected to abuse, not simply when there is some chance for future abuse of a child (p. 56).

Of course, this commentary is confined to the US context. In other countries, there is a legal duty to warn about the potential for harm is a specific group of people are at risk (e.g., a school class), and so Walker et al.’s (2022) observations are not applicable universally. However, they do highlight the ethical conundrum facing professionals who are trying to make ethically (and, in jurisdictions whereby reporting actual or suspected abuse is mandated, legally) defensible decisions while maintaining a safe and trusting therapeutic environment for patients to explore any issues that are troubling to them (Beggs Christofferson, 2019; Lievesley & Harper, 2021). This is mirrored in our data. Again, we highlight the importance of education (for student professionals) and ongoing training (for those already qualified) to construct guidelines that identify concrete factors that would determine the need to report. Examples might include disclosures of spending time or engaging with a specific child in a context that it would not be expected (be this online or offline). This process of forming explicit guidance also necessitates a degree of cooperation with professional bodies and legislators to ensure that there are no sanctions brought against professionals who, in the absence of such concrete indicators of abuse, fail to report a patient who goes on to commit an offense. Such cooperation would reduce cognitive load on professionals and allow them the headspace to make therapeutically informed decisions about breaking confidentiality within a clearly defined reporting context.

### Limitations and Future Directions

Although readers may interpret our analyses as confirmatory, our intention in this work was to explore the current state of general healthcare knowledge and practice in relation to working with people who disclose sexual attractions to children. As such, our work was not theoretically driven, nor did we seek to test specific hypotheses in this project. However, the advantage of using exploratory designs is in the generation of new research questions and testable hypotheses. We have posited some such future avenues for research here, including comparisons between general and specialist healthcare providers in their levels of comfort, willingness to treat, and treatment approaches and targets, and in the design of effective training programs. Other research teams may wish to build on this work when designing studies within their own jurisdictions, or when exploring healthcare provision for MAPs in other geographical or logistical contexts.

Related to our use of inferential statistics, some readers may question our choice to set our alpha level at the standard threshold of 0.05, rather than a more conservative 0.005, given the number of analyses ran in this work (for a discussion of justifying alpha levels, see Lakens et al., 2018). We share field-wide concerns about the replicability of social science research, and therefore encourage caution when interpreting results in this paper where *p*-values are below, but close to, the 0.05 threshold for significance. However, we also stand behind these results when taking them in conjunction with their associated effect sizes. Most of our statistically significant findings occur with a *p*-value of < 0.001 and therefore would remain interpretable as such if a reader chose to use *p* < 0.005 are their threshold. Of those where 0.005 < *p* < 0.05, effect sizes range from *d* = 0.31–0.45. In standard terms, these effects are small-to-moderate in magnitude (Kelley & Preacher, 2012), but in social science terms are moderate-to-large in terms of absolute size, irrespective of the associated *p*-value (Funder & Ozer, 2019; Gignac & Szodorai, 2016; Lovakov & Agadullina, 2021).

Our use of a convenience sampling technique limits the extent to which our findings can be generalized to the broader healthcare context. However, in exploratory research, generalization is not a primary goal. Here, we sought to provide an overview of current healthcare practices when a patient discloses sexual attractions to children. This first step is an important advance in our understanding of existing beliefs and the relationships between beliefs and decision-making in primary healthcare settings. To reduce the effect of local reporting or confidentiality laws, we only recruited participants from countries whereby reporting guidelines set out in professional codes or legal statutes were limited to cases involving actual or suspected abuse. That is, in the countries represented here, professionals may report service users if they have evidence of abuse taking place, or if they perceive a meaningful risk of child abuse, though as previously discussed “meaningful risk” is a relative decision made at the individual level. We do not know how reporting practices or professional decision-making might change in contexts whereby confidentiality must be upheld even when illegal behavior is disclosed (e.g., Germany). Exploring these issues, and the moral strains that they may have on professionals, is an important future consideration.

Related to our sampling approach, self-selection appears to have been a significant issue. This is particularly the case when considering the relative samples sizes from the UK vs. all other countries. We do not have a particularly strong reason for this disparity in sample size, save for our own geographical location being in the UK. As such, it may have been that professionals working in other jurisdictions assumed a lack of relevance to their work. This possibility is strengthened by the comment of an anonymous reviewer of an earlier iteration of this work, who suggested that our use of “safeguarding team” (rather than “child protection hotline”, for example) may have been confusing for North American respondents. Similarly, there may be more clearly defined referral and treatment responsibilities of different professional groups. For example, nurses in the UK have referral obligations where their interactions with patients necessitate this, whereas in other jurisdictions this does not appear to be the case. This may therefore lead to some cases where our items about professional practice were not relevant to a small number of participants. These issues highlight the importance of establishing broad and geographically disperse networks of researchers working collaboratively on projects to both ensure the applicability the research materials and boost external perception of relevance to potential participants.

In this work we used self-report measures throughout, which may be seen as a limitation of our methods. Although the use of self-report methods is commonplace in social science research generally, and in work on professional views about support for MAPs (Beggs Christofferson, 2019; Goodier & Lievesley, 2018; Jahnke et al., 2015a, b; Levenson & Grady, 2019a; Parr & Pearson, 2019; Stephens et al., 2021; Walker et al., 2022), exploring actual behavior when working with this population is an important future research approach. That is, self-reports are inherently subject to self-presentation biases (Paulhus & Vazire, 2007), especially when one’s personal and collective professional practice is under scrutiny. Recollections may also be subject to hindsight bias for similar reasons (Bernstein et al., 2016). One potential method of collecting practice-based data would be to trial a decision-making tool that allows professionals to log each patient encounter, along with information about presenting problems, feelings of professional competence, and referral decisions. Enacting such a process of regular data collection might also reduce the extent to which our own sampling was subject to another layer of self-selection bias related to the perceived relevance of the study as a function of whether a potential participant has ever had a patient disclose sexual attractions to children. That is, alongside patient-by-patient reporting, prospective designs might look to collect data on a monthly or quarterly basis to track perceptions and beliefs about this population over time in response to the exposure (or lack thereof) to disclosures.

Related to our measures, some of these used “sexual interests/attractions to children” as the reference group, and others used “pedophiles/pedophilia”. This is a limitation to the study owing to the fact that we know people make intuitive attributions about the “pedophile” label, leading to a divergence in responses to this term (Imhoff, 2015; King & Roberts, 2017). Our choice of measures was driven by a desire to use validated scales of the constructs of interest, and as such there appears to be a need to standardize the language used in this area of scholarship. Recent work uses labels such as “pedophiles” (Harper et al., 2018, 2021; Imhoff, 2015; Jahnke et al., 2015a, b), “minor-attracted persons” (Grady & Levenson, 2019; Grady et al., 2020; Levenson & Grady, 2019b), “people with pedohebephilia” (Martijn et al., 2020), and “child-attracted persons” (Martijn et al., 2021). These terms will undoubtedly all be accompanied by their own unique set of attributions, and a useful next step in the research process would be to standardize the language used to produce measures that can facilitate reproducible research.

## Conclusions

In this work, we have presented an initial snapshot of current non-specialist healthcare professionals’ views, experiences, and decision-making processes about working with MAPs. Our data highlight a number of misconceptions held by healthcare professionals about this population and the nature of their attractions, including in relation to fundamental definitions of pedophilia and its etiology. We have also demonstrated potential incongruences between professional and MAPs’ treatment priorities. There is a clear and demonstrable need for awareness-raising in primary healthcare settings to improve professionals’ knowledge, comfort, and confidence in working with disclosures of sexual attractions to children in order to facilitate the development of responsive and effective support services in the non-offending or prevention context.

## Supplementary Information

Below is the link to the electronic supplementary material.

Supplementary file1 (PDF 302 KB)

## Data Availability

Consent was not obtained from participants to share their data during the informed consent procedure, as there are concerns about participant identification. As such, data are not available for dissemination.

## Footnotes

1 Nine organizations responded favorably to our request, although we did not receive confirmation about whether the survey had been distributed, nor to how many organizational members.

## References

[CR1] Allardyce S, Lievesley R, Hocken K, Elliott H, Winder B, Blagden N, Banyard P (2018). Theories of sexual crime prevention. Sexual crime and prevention.

[CR2] Arnow BA, Steidtmann D (2014). Harnessing the potential of the therapeutic alliance. World Psychiatry.

[CR3] B4U-ACT. (2011). *Mental health care & professional literature*. https://www.b4uact.org/research/survey-results/spring-2011-survey/

[CR4] B4U-ACT. (2017). *Principles and perspectives of practice*. http://www.b4uact.org/about-us/principles-and-perspectives-of-practice/

[CR5] Bailey JM (2015). A failure to demonstrate changes in sexual interest in pedophilic men: Comment on Müller et al. (2014). Archives of Sexual Behavior.

[CR6] Bailey JM, Hsu KJ, Bernhard PA (2016). An Internet study of men sexually attracted to children: Sexual attraction patterns. Journal of Abnormal Psychology.

[CR7] Beggs Christofferson SM (2019). Is preventive treatment for individuals with sexual interest in children viable in a discretionary reporting context?. Journal of Interpersonal Violence.

[CR8] Beier KM, Neutze J, Mundt IA, Ahlers CJ, Goecker D, Konrad A, Schaefer GA (2009). Encouraging self-identified pedophiles and hebephiles to seek professional help: First results of the Prevention Project Dunkelfeld (PPD). Child Abuse and Neglect.

[CR9] Bernstein DM, Aßfalg A, Kumar R, Ackerman R, Dunlosky J, Tauber SK (2016). Looking backward and forward on hindsight bias. The Oxford handbook of metamemory.

[CR10] Blanchard R (2010). The DSM diagnostic criteria for pedophilia. Archives of Sexual Behavior.

[CR11] Blanchard R, Lykins AD, Wherrett D, Kuban ME, Cantor JM, Blak T, Dickey R, Klassen PE (2009). Pedophilia, hebephilia, and the DSM-V. Archives of Sexual Behavior.

[CR12] Browne J, Mueser KT, Meyer-Kalos P, Gottlieb JD, Estroff SE, Penn DL (2019). The therapeutic alliance in individual resiliency training for first episode psychosis: Relationship with treatment outcomes and therapy participation. Journal of Consulting and Clinical Psychology.

[CR13] Cantor JM (2015). Purported changes in pedophilia as statistical artefacts: Comment on Müller et al. (2014). Archives of Sexual Behavior.

[CR14] Cantor JM, Blanchard R, Christensen BK, Dickey R, Klassen PE, Beckstead AL, Blak T, Kuban ME (2004). Intelligence, memory, and handedness in pedophilia. Neuropsychology.

[CR15] Cantor JM, Kabani N, Christensen BK, Zipursky RB, Barbaree HE, Dickey R, Klassen PE, Mikulis DJ, Kuban ME, Blak T, Richards BA, Hanratty MK, Blanchard R (2008). Cerebral white matter deficiencies in pedophilic men. Journal of Psychiatric Research.

[CR16] Cantor JM, Lafaille S, Soh DW, Moayedi M, Mikulis DJ, Girard TA (2015). Diffusion tensor imaging of pedophilia. Archives of Sexual Behavior.

[CR17] Cantor JM, McPhail IV (2016). Non-offending pedophiles. Current Sexual Health Reports.

[CR18] Christiansen C, Martinez-Dettamanti M, Lievesley R, Hocken K, Elliott H, Winder B, Blagden N, Banyard P (2018). Prevention in action: Exploring prevention initiatives and current practices. Sexual crime and prevention.

[CR19] Clegg M, Pye J, Wylie KR (2016). Undergraduate training in human sexuality—Evaluation of the impact on medical doctors’ practice ten years after graduation. Sexual Medicine.

[CR20] Cohen LJ, Wilman-Depena S, Barzilay S, Hawes M, Yaseen Z, Galynker I (2020). Correlates of chronic suicidal ideation among community-based minor-attracted persons. Sexual Abuse.

[CR21] Eher R, Olver ME, Heurix I, Schilling F, Rettenberger M (2015). Predicting reoffense in pedophilic child molesters by clinical diagnoses and risk assessment. Law and Human Behavior.

[CR22] Elchuk DL, McPhail IV, Olver ME (2022). Stigma-related stress, complex correlates of disclosure, mental health, and loneliness in minor-attracted people. Stigma and Health.

[CR23] Elvins R, Green J (2008). The conceptualization and measurement of therapeutic alliance: An empirical review. Clinical Psychology Review.

[CR24] Feldman DB, Crandall CS (2007). Dimensions of mental illness stigma: What about mental illness causes social rejection?. Journal of Social and Clinical Psychology.

[CR26] Finkelhor D (1984). Child sexual abuse: New theory and research.

[CR27] Funder DC, Ozer DJ (2019). Evaluating effect size in psychological research: Sense and nonsense. Advances in Methods and Practices in Psychological Science.

[CR28] Gannon T, Terriere R, Leader T (2012). Ward and Siegert’s pathways model of child sexual offending: A cluster analysis evaluation. Psychology, Crime & Law.

[CR29] Gignac GE, Szodorai ET (2016). Effect size guidelines for individual differences researchers. Personality and Individual Differences.

[CR30] Goodier S, Lievesley R (2018). Understanding the needs of individuals at risk of perpetrating child sexual abuse: A practitioner perspective. Journal of Forensic Psychology Research and Practice.

[CR31] Grady MD, Levenson JS, Mesias G, Kavanagh S, Charles J (2019). “I can’t talk about that”: Stigma and fear as barriers to preventive services for minor-attracted persons. Stigma and Health.

[CR32] Green SB (1991). How many subjects does it take to do a regression analysis?. Multivariate Behavioral Research.

[CR33] Grundmann D, Krupp J, Scherner G, Amelung T, Beier KM (2016). Stability of self-reported arousal to sexual fantasies involving children in a clinical sample of pedophiles and hebephiles. Archives of Sexual Behavior.

[CR34] Grundmann D, Krupp J, Scherner G, Amelung T, Beier KM (2017). Response to Tozdan and Briken's (2016) "Accepting sexual interest in children as unchangeable: One claim fits for all?" [Letter to the Editor]. Archives of Sexual Behavior.

[CR35] Harper CA, Bartels RM, Hogue TE (2018). Reducing stigma and punitive attitudes toward pedophiles through narrative humanization. Sexual Abuse.

[CR36] Harper CA, Lievesley R, Blagden NJ, Hocken K (2021). Humanizing pedophilia as stigma reduction: A large-scale intervention study. Archives of Sexual Behavior.

[CR37] Hocken K, Lievesley R, Hocken K, Elliott H, Winder B, Blagden N, Banyard P (2018). Safer living foundation: The Aurora project. Sexual crime and prevention.

[CR38] Horvath AO, Del Re AC, Flückiger C, Symonds D (2011). Alliance in individual psychotherapy. Psychotherapy.

[CR39] Houtepen JABM, Sijtsema JJ, Bogaerts S (2016). Being sexually attracted to minors: Sexual development, coping with forbidden feelings, and relieving sexual arousal in self-identified pedophiles. Journal of Sex & Marital Therapy.

[CR40] Jahnke S (2018). The stigma of pedophilia: Clinical and forensic implications. European Psychologist.

[CR41] Jahnke S, Philipp K, Hoyer J (2015). Stigmatizing attitudes towards people with pedophilia and their malleability among psychotherapists in training. Child Abuse & Neglect.

[CR42] Jahnke S, Schmidt AF, Geradt M, Hoyer J (2015). Stigma-related stress and its correlates among men with pedophilic sexual interests. Archives of Sexual Behavior.

[CR43] Jahnke S, Schmitt S, Malón A (2018). What if the child appears to enjoy it? Moral attitudes toward adult–child sex among men with and without pedohebephilia. Journal of Sex Research.

[CR44] Jones SJ, Ó Ciardha C, Elliott IA (2021). Identifying the coping strategies of nonoffending pedophilic and hebephilic individuals from their online forum posts. Sexual Abuse.

[CR45] Kelley K, Preacher KJ (2012). On effect size. Psychological Methods.

[CR46] Konrad A, Haag S, Scherner G, Amelung T, Beier KM (2017). Previous judicial detection and paedophilic sexual interest partially predict psychological distress in a non-forensic sample of help-seeking men feeling inclined to sexually offend against children. Journal of Sexual Aggression.

[CR47] Lakens D, Adolfi FG, Albers CJ, Anvari F, Apps MAJ, Argamon SE, Baguley T, Becker RB, Benning SD, Bradford DE, Buchanan EM, Caldwell AR, Van Calster B, Carlsson R, Chen S-C, Chung B, Colling LJ, Collins GS, Crook Z, Cross ES, Daniels S (2018). Justify your alpha. Nature Human Behaviour.

[CR48] Lawrence AL, Willis GM (2021). Understanding and challenging stigma associated with sexual interest in children: A systematic review. International Journal of Sexual Health.

[CR49] Levenson JS, Grady MD (2019). “I could never work with those people…”: Secondary prevention of child sexual abuse via a brief training for therapists about pedophilia. Journal of Interpersonal Violence.

[CR50] Levenson JS, Grady MD (2019). Preventing sexual abuse: Perspectives of minor-attracted persons about seeking help. Sexual Abuse.

[CR51] Levenson JS, Grady MD, Morin JW (2020). Beyond the “ick factor”: Counseling non-offending persons with pedophilia. Clinical Social Work Journal.

[CR52] Levenson JS, Willis GM, Vicencio CP (2017). Obstacles to help-seeking for sexual offenders: Implications for prevention of sexual abuse. Journal of Child Sexual Abuse.

[CR53] Lievesley, R., Elliott, H., Blagden, N., & Winder, B. (2016). *Resist not desist: A retrospective exploration of viable prevention strategies helping individuals to avoid committing their first sexual offence against a child*. Paper presented at the International Association for the Treatment of Sexual Offenders, Copenhagen, Denmark.

[CR54] Lievesley R, Elliott H, Hocken K, Lievesley R, Hocken K, Elliott H, Winder B, Blagden N, Banyard P (2018). Future Directions: Moving forward with sexual crime prevention. Sexual crime and prevention.

[CR55] Lievesley R, Harper CA (2021). Applying desistance principles to improve wellbeing and prevent child sexual abuse among minor-attracted persons. Journal of Sexual Aggression.

[CR56] Lievesley R, Harper CA, Elliott H (2020). The internalization of social stigma among minor-attracted persons: Implications for treatment. Archives of Sexual Behavior.

[CR57] Locati F, Rossi G, Parolin L (2019). Interactive dynamics among therapist interventions, therapeutic alliance and metacognition in the early stages of the psychotherapeutic process. Psychotherapy Research.

[CR58] Lovakov A, Agadullina ER (2021). Empirically derived guidelines for effect size interpretation in social psychology. European Journal of Social Psychology.

[CR59] Malone L, Jeglic EL, Calkins C (2016). Help wanted: Young pedophiles and the importance of primary prevention. Sexual violence.

[CR60] Marshall WL (2010). The role of attachments, intimacy, and loneliness in the etiology and maintenance of sexual offending. Sexual and Relationship Therapy.

[CR61] Martijn FM, Babchishin KM, Pullman LE, Seto MC (2020). Sexual attraction and falling in love in persons with pedohebephilia. Archives of Sexual Behavior.

[CR62] McPhail IV, Stephens S, Heasman A (2018). Legal and ethical issues in treating clients with pedohebephilic interests. Canadian Psychology.

[CR63] Mokros A, Banse R (2019). The “dunkelfeld” project for self-identified pedophiles: A reappraisal of its effectiveness. Journal of Sexual Medicine.

[CR64] Müller K, Curry S, Ranger R, Briken P, Bradford J, Fedoroff JP (2014). Changes in sexual arousal as measured by penile plethysmography in men with pedophilic sexual interest. Journal of Sexual Medicine.

[CR65] Mundfrom DJ, Shaw DG, Ke TL (2005). Minimum sample size recommendations for conducting factor analyses. International Journal of Testing.

[CR66] Nienhuis JB, Owen J, Valentine JC, Black SW, Halford TC, Parazak SE, Budge S, Hilsenroth M (2018). Therapeutic alliance, empathy, and genuineness in individual adult psychotherapy: A meta-analytic review. Psychotherapy Research.

[CR67] Parr J, Pearson D (2019). Non-offending minor-attracted persons: Professional practitioners’ views on the barriers to seeking and receiving their help. Journal of Child Sexual Abuse.

[CR68] Paulhus DL, Vazire S, Robins RW, Fraley RC, Krueger RF (2007). The self-report method. Handbook of research methods in personality psychology.

[CR70] Schmidt AF, Mokros A, Banse R (2013). Is pedophilic sexual preference continuous? A taxometric analysis based on direct and indirect measures. Psychological Assessment.

[CR71] Seto MC (2009). Pedophilia. Annual Review of Clinical Psychology.

[CR72] Seto MC (2012). Is pedophilia a sexual orientation?. Archives of Sexual Behavior.

[CR73] Seto MC (2017). The puzzle of male chronophilias. Archives of Sexual Behavior.

[CR74] Seto MC (2019). The motivation-facilitation model of sexual offending. Sexual Abuse.

[CR75] Shields RT, Murray SM, Ruzicka AE, Buckman C, Kahn G, Benelmouffok A, Letourneau EJ (2020). Help wanted: Lessons on prevention from young adults with a sexual interest in prepubescent children. Child Abuse & Neglect.

[CR76] Stephens S, Cantor JM, Goodwill AM, Seto MC (2017). Multiple indicators of sexual interest in prepubescent or pubescent children as predictors of sexual recidivism. Journal of Consulting and Clinical Psychology.

[CR77] Stephens S, McPhail IV, Heasman A, Moss S (2021). Mandatory reporting and clinician decision-making when a client discloses sexual interest in children. Canadian Journal of Behavioural Science / Revue Canadienne des Sciences du Comportement.

[CR78] Stephens S, Seto MC, Phenix A, Hoberman HM (2018). Hebephilic sexual offending. Sexual offending: Predisposing antecedents, assessments and management.

[CR79] Stephens S, Seto MC, Cantor JM, Lalumière ML (2019). The revised Screening Scale for Pedophilic Interests (SSPI-2) may be a measure of pedohebephilia. Journal of Sexual Medicine.

[CR80] Stephens S, Seto MC, Goodwill AM, Cantor JM (2017). Evidence of construct validity in the assessment of hebephilia. Archives of Sexual Behavior.

[CR81] Stephens S, Seto MC, Goodwill AM, Cantor JM (2018). Age diversity among victims of hebephilic sexual offenders. Sexual Abuse.

[CR82] Stevens E, Wood J (2019). “I despise myself for thinking about them”: A thematic analysis of the mental health implications and employed coping mechanisms of self-reported non-offending minor attracted persons. Journal of Child Sexual Abuse.

[CR83] Stott DB (2013). The training needs of general practitioners in the exploration of sexual health matters and providing sexual healthcare to lesbian, gay and bisexual patients. Medical Teacher.

[CR84] Tenbergen G, Wittfoth M, Frieling H, Ponseti J, Walter M, Walter H, Beier KM, Schiffer B, Kruger THC (2015). The neurobiology and psychology of pedophilia: Recent advances and challenges. Frontiers in Human Neuroscience.

[CR85] Tozdan S, Briken P (2017). Accepting sexual interest in children as unchangeable: One claim fits for all? Comment on Grundman, Krupp, Scherner, Amelung, and Beier's (2016) “Stability of self-reported arousal to sexual fantasies involving children in a clinical sample of pedophiles and hebephiles” [Letter to the Editor]. Archives of Sexual Behavior.

[CR86] Ucsnik L, Kottmel A, Körbel TH, Bitzer J, Teleky B, Marktl W (2018). 567 Do doctors specialising for training in health ressorts integrate sexual health problems in daily patient-managment _ self-assessment results at the annual training-course for doctors in health resorts - Bad Gastein, March 2017 [Abstract]. Journal of Sexual Medicine.

[CR87] VanVoorhis CRW, Morgan BL (2007). Understanding power and rules of thumb for determining sample sizes. Tutorials in Quantitative Methods for Psychology.

[CR88] Walker A, Butters RP, Nichols E (2022). “I would report it even if they have not committed anything”: Social service students’ attitudes toward minor-attracted people. Sexual Abuse.

[CR89] Ward T, Beech A (2006). An integrated theory of sexual offending. Aggression and Violent Behavior.

[CR90] Ward T, Siegert RJ (2002). Toward a comprehensive theory of child sexual abuse: A theory knitting perspective. Psychology, Crime & Law.

[CR91] Wielinga F, Margeotes K, Olver ME (2021). Clinical and risk relevance of intimacy and loneliness in a treated sample of men who have offended sexually. Journal of Sexual Aggression.

