# Supplementary material for: Primary Health Professionals’ Beliefs, Experiences, and Willingness to Treat Minor-Attracted Persons
Source: Arch Sex Behav. 2022 Jan 27;51(2):923–43. doi: 10.1007/s10508-021-02271-7 (PMC8793822; doi:10.1007/s10508-021-02271-7)
Supplement: Supplementary file 1 — Supplementary file1 (PDF 302 KB) [file 10508_2021_2271_MOESM1_ESM.pdf]

Supplementary Material – Analysis of Semantic Attributions about Sexual Interest in Children

Context

We adapted Feldman & Crandall's (2007) 17-item measure of attributions about mental health to tap into attributions that participants made about the nature of "sexual interests in children". Each item (e.g., "To what extent are the clinical symptoms experienced by people with sexual interests in children completely sexual in nature?") was rated using a seven-point slider, with each item using semantically appropriate anchors for the extreme values of 1 and 7. We wanted to explore the dimensionality of this measure within the Results section of our paper, and then to look at professionals' attributions about the nature of sexual interests in children. The exact wording and anchoring of these items were as follows:

Please answer the following questions with reference to sexual interests in children:

1. *Not at all dangerous to others / Very dangerous to others*
2. *Symptoms are the person's fault / Symptoms are not the person's fault*
3. *Disorder is avoidable / Disorder is unavoidable*
4. *Person is completely in touch with reality / Person is completely out of touch with reality*
5. *Quite common / Very rare*
6. *Not at all disruptive in social situations / Very disruptive*
7. *Not treatable with medication / Treatable with medication*
8. *Causes problems at work / Does not cause problems at work*
9. *Not embarrassing to have / Embarrassing to have*
10. *Symptoms are not sexual in nature / Symptoms are very sexual in nature*
11. *Acute (short–lived) without treatment / Chronic (long–lasting) without treatment*
12. *Person is in complete control of him/herself / Person is completely unable to control him/herself*
13. *Mild / Severe*
14. *Not treatable with psychotherapy / Treatable with psychotherapy*
15. *One sex gets it / Both sexes get it*
16. *Potentially concealable / Publicly visible*
17. *Not hereditary or genetic / Hereditary or genetic*

After conducting exploratory analyses of the dimensionality of this measure, no meaningful factors underpinned the data, and as such we presented between-groups comparisons of each item. However, a reviewer of this earlier version suggested removing this owing to the degree of overlap between the attribution scale's items and those contained within the SPS (Imhoff, 2015). As such, these analyses are not reported within the main body of this paper. In the interest of transparency, though, we present them within this supplement.

Results

We subjected the attributions measure to an exploratory factor analysis to explore whether participants made attributions about MAPs in a way that could be conceived of as belief clusters. This analysis yielded adequate outcomes for the Kaiser-Meyer-Olkin coefficient (0.71) and Bartlett's test for sphericity was significant, $\chi^2(136) = 436.62$, $p < .001$. Taken in combination, these tests show that the data we collected were suitable for factor analysis. However, the rotated pattern matrix (using an oblique 'direct oblimin' rotation method and extracting factors with eigenvalues greater than 1 using the maximum likelihood method of factor extraction) produced seven factors that each contained either one (four factors) or two (three factors) significantly-loading items when considering Field's (2005) cut-off item loading value of 0.40 (see Table S1). As this does not meaningfully reduce the measure into a smaller number of distinct domains, we present descriptive statistics for each item, broken down by professional group, in Table S2.

Table S1. Item loadings from the exploratory factor analysis of the adapted attributions measure.

	Factor						
	1	2	3	4	5	6	7
How severe are the clinical symptoms associated with having sexual interests in children?	**.973**	-.020	.019	.057	.037	.057	-.077
To what extent are sexual interests in children avoidable?	.016	**.964**	-.081	.051	-.030	-.105	.057
To what extent are sexual interests in children the person's fault?	-.104	**.619**	-.026	.176	.039	.251	-.131
To what extent are sexual interests in children treatable with psychotherapy?	.054	.212	.122	-.081	.039	-.051	.097
To what extent are sexual interests in children treatable with medication?	.021	-.092	**.847**	.039	-.101	.010	.086
To what extent do people with sexual interests in children have problems at work?	.077	.204	.318	.069	.216	-.018	-.125
How dangerous are people with sexual interests in children?	-.003	.320	.066	**.528**	-.053	.127	-.275
How socially disruptive are people with sexual interests in children?	.028	.211	.144	**.512**	.045	.138	-.071
To what extent are sexual interests in children acute or chronic without treatment?	.275	-.021	-.021	**.374**	.089	-.065	.022
To what extent are sexual interests in children embarrassing to have?	.077	-.030	-.092	.021	**.762**	.006	.135
To what extent are the clinical symptoms experienced by people with sexual interests in children completely sexual in nature?	-.054	.091	.329	.059	**.350**	.132	-.151
How common are sexual interests in children?	-.043	-.041	-.070	.053	.154	**.688**	.014
To what extent are sexual interests in children genetically heritable?	.055	.031	.114	.027	-.097	**.421**	-.034
To what extent are people with sexual interests in children in touch with reality?	-.016	-.111	-.134	.080	-.158	.244	.244
To what extent are sexual interests in children experienced by both sexes?	.084	.020	.015	-.120	-.051	.169	.032
To what extent are people with sexual interests in children able to control themselves?	-.016	.063	.045	-.102	.111	.057	**.651**
How visible are sexual interests in children?	.081	.082	-.048	-.276	.071	.256	**-.347**

Table S2. Beliefs and attributions about patients with sexual attractions to children, by professional group

	**Professional group**		
Statement	Primary medical	Mental health	Inferential statistics
How dangerous are people with sexual interests in children?	5.29 (1.35)	4.61 (1.22)	$t(160) = 3.35$, $p = .001$, $d = 0.53$
To what extent are sexual interests in children the person's fault?	3.19 (1.55)	2.26 (1.29)	$t(154) = 4.11$, $p < .001$, $d = 0.66$
To what extent are sexual interests in children avoidable?	3.90 (1.63)	3.33 (1.45)	$t(155) = 2.30$, $p = .023$, $d = 0.37$
To what extent are people with sexual interests in children in touch with reality?	3.91 (1.58)	4.30 (1.56)	$t(157) = -1.56$, $p = .121$, $d = -0.25$
How common are sexual interests in children?	3.23 (1.33)	3.36 (1.15)	$t(157) = -0.65$, $p =.520$, $d = -0.10$
How socially disruptive are people with sexual interests in children?	4.30 (1.82)	3.90 (1.62)	$t(157) = 1.45$, $p = .148$, $d = 0.23$
To what extent are sexual interests in children treatable with medication?	2.70 (1.26)	2.58 (1.27)	$t(147) = 0.58$, $p = .563$, $d = 0.09$
To what extent do people with sexual interests in children have problems at work?	3.89 (1.62)	3.33 (1.31)	$t(150) = 2.38$, $p = .019$, $d = 0.39$
To what extent are sexual interests in children embarrassing to have?	6.11 (1.05)	5.86 (1.33)	$t(156) = 1.33$, $p = .186$, $d = 0.21$
To what extent are the clinical symptoms experienced by people with sexual interests in children completely sexual in nature?	3.75 (1.33)	3.19 (1.22)	$t(154) = 2.76$, $p = .007$, $d = 0.44$
To what extent are sexual interests in children acute or chronic without treatment?	5.47 (1.23)	5.59 (1.17)	$t(157) = -0.63$, $p = .531$, $d = -0.10$
To what extent are people with sexual interests in children able to control themselves?	4.08 (1.24)	4.46 (1.08)	$t(157) = -2.10$, $p = .037$, $d = -0.33$
How severe are the clinical symptoms associated with having sexual interests in children?	4.49 (1.27)	4.62 (1.17)	$t(155) = -0.63$, $p = .532$, $d = -0.10$
To what extent are sexual interests in children treatable with psychotherapy?	4.63 (1.13)	4.28 (1.09)	$t(158) = 1.99$, $p = .049$, $d = 0.31$
To what extent are sexual interests in children experienced by both sexes?	4.54 (1.83)	4.38 (1.57)	$t(157) = 0.63$, $p = .533$, $d = 0.10$
How visible are sexual interests in children?	1.86 (1.10)	1.77 (1.12)	$t(147) = 0.50$, $p = .621$, $d = 0.08$
To what extent are sexual interests in children genetically heritable?	2.33 (1.22)	2.08 (1.27)	$t(140) = 1.20$, $p = .231$, $d = 0.20$

Note. Mean values range from 1-7 with standard deviations in parentheses. Higher scores indicate an answer that equates to a ‘very’ or completely’ response to the question. For questions containing two options, higher scores indicate responding in the affirmative to the latter option. Effect sizes show how those working in primary medical contexts scored in comparison to those working within mental health settings.

In comparison to professionals working in mental health, those working in a primary medical setting (e.g., general practitioners, nurses, and medical physicians) were more likely than mental health professionals to view patients with sexual attractions to children as being dangerous, attribute blame to patients for their attractions, and see having sexual attractions to children as being avoidable. They were also more likely to think that MAPs would have issues with work, view clinical symptoms as being confined to the sexual domain, and support the use of psychotherapy as the most effective treatment option. Medical professionals were also less likely than mental health professionals to say that MAPs could control their behavior.

Supplementary Material – Professionals' Knowledge about Pedophilia

We recoded each myth / knowledge statement within our dataset to be scored 0 = incorrect and 1 = correct. Following this, we computed a 'proportion correct' score for each participant (labeled 'knowledge accuracy'). This outcome ranged from 0 to 1 where higher scores indicated greater factual knowledge about pedophilia. Descriptive statistics for each statement, broken down by professional group, are presented in Table S3.

Table S3. Knowledge about pedophilia, by professional group

	Professional group		
Statement	Primary medical	Mental health	Inferential statistics
Most convicted child abusers are pedophiles	0.68 (0.47)	0.78 (0.42)	χ^2 (1) = 2.12, p = .146, φ = 0.11
Some pedophiles can have fulfilling sexual relationships with adult partners	0.85 (0.36)	0.88 (0.33)	χ^2 (1) = 0.24, p = .625, φ = 0.04
Pedophiles do not choose to be attracted to children	0.66 (0.48)	0.82 (0.39)	χ^2 (1) = 5.53, p = .019, φ = 0.18
Pedophilia is a sexual attraction to children below the age of 16	0.26 (0.44)	0.35 (0.48)	χ^2 (1) = 1.70, p = .192, φ = 0.10
All convicted child abusers are pedophiles	0.87 (0.34)	0.93 (0.26)	χ^2 (1) = 1.71, p = .191, φ = 0.10
Pedophiles choose to be attracted to children	0.91 (0.28)	1.00 (0.00)	χ^2 (1) = 7.40, p = .007, φ = 0.21
Less than half of child abusers are pedophiles	0.46 (0.50)	0.42 (0.50)	χ^2 (1) = 0.29, p = .589, φ = 0.04
Pedophilia is a sexual attraction to children below the age of 11	0.23 (0.43)	0.43 (0.50)	χ^2 (1) = 7.58, p = .006, φ = 0.21
Pedophiles always act on their sexual urges	0.89 (0.32)	1.00 (0.00)	χ^2 (1) = 9.64, p = .002, φ = 0.24
Overall accuracy	0.65 (0.22)	0.74 (0.17)	t(163) = -2.91, p = .004, d = -0.45

Note. Values range from 0-1 and represent mean levels of accuracy within each group with standard deviations in parentheses. Scores closer to 1 indicate greater levels of accuracy (i.e., multiplying by 100 will indicate an average % correct score). The 'overall accuracy' test was computed by converting the % correct score for each participant into a continuous *z*-score and comparing the two groups using this *z*-scores as the dependent variable. Statements in italics are coded as false based on the current empirical evidence. Effect sizes show how those working in primary medical contexts scored in comparison to those working within mental health settings.

Supplementary Material – Exploratory Factor Analysis of the Treatment Priorities Measure

Because the treatment priorities measure has not been used in peer-reviewed research before, we initially conducted an exploratory factor analysis to examine its dimensionality. Within this analysis we used the maximum likelihood method of factor extraction and an oblique (direct oblimin) rotation to allow any underlying factors to correlate with each other. We retained factors with an eigenvalue greater than one, with participants with missing values excluded in a pairwise manner. The Kaiser-Meyer-Olkin measure of sampling adequacy was calculated as 0.76, and Bartlett's test of sphericity was significant, $\chi^2(55) = 682.20$, $p < .001$. These statistics indicate that the data collected on the treatment priorities scale were suitable for factor analysis.

Three factors were retained (Table S4). The first factor was comprised of two items and relate to general mental health concerns. The second factor was much larger, consisting of five items related to the forensic control of sexual attractions. The final factor contained three items that were related to living within society with a stigmatized sexual attraction pattern. When considering Field's (2005) factor loading cut-off value of 0.40, one item ("To help the patient deal with sexual frustration") loaded significantly on to both factor two and factor three. As such, we excluded this item from the measure. An average score for each factor was computed and used in subsequent exploratory analyses.

Table S4. Pattern matrix for the exploratory factor analysis of the treatment priorities measure

	**Treatment target**		
	General mental health concerns	Forensic control of sexual attractions	Living with stigmatized attractions
To help the patient feel happier or at peace	**1.016**	-.105	.061
To treat the patient's general mental health concerns in relation to their sexual attraction (e.g., depression or anxiety)	**.528**	.154	.111
To help the patient to understand the cause of their attraction	-.038	**.741**	.244
To help the patient to develop or increase an attraction to adults	.042	**.632**	-.100
For me to understand the cause of the patient's attraction	-.025	**.605**	.025
To help the patient control their sexual feelings	-.001	**.524**	.149
To help the patient to extinguish or reduce an attraction to children	.136	**.430**	-.258
To help the patient deal with society's negative response to their sexual attraction	-.023	.062	**.626**
To help the patient figure out how to live in society with their sexual attraction	.192	-.083	**.615**
To improve the patient's self-concept	.198	.086	**.546**
To help the patient deal with their sexual frustration	.060	.460	.494
M (*SD*)	8.62 (1.45)	7.42 (1.72)	6.69 (2.05)
α	.73	.73	.70

Note. Items with loadings in **bold typeface** were retained.

The three treatment target factors were all positively correlated with each other, though to a small-to-moderate degree (see Table S5). This differentiation between treatment targets is supportive of the view that there are different clusters of needs that might be targeted by healthcare professionals when working with MAPs. A summary of the descriptive statistics indicating treatment prioritization scores between the two professional groups is presented in Table S6.

Table S5. Zero-order correlations between treatment target prioritization

Treatment factor	1	2	3
1. Mental health concerns	-		
2. Controlling sexual attractions	.33	-	
3. Living with stigmatized attractions	.44	.27	-

Note. All correlations are statistically significant ($p < .001$).

Table S6. Treatment prioritization scores, by professional group

	**Professional group**	
Treatment factor	Primary medical	Mental health
Mental health concerns	8.82 (1.26)	8.42 (1.58)
Controlling sexual attractions	7.22 (1.66)	7.70 (1.62)
Living with stigmatized attractions	6.48 (2.12)	6.82 (1.79)

Note. Figures represent mean values with standard deviations in parentheses. Possible score ranges from 1-10, with higher values indicating greater levels of prioritization.

Supplementary Material – Exploratory Analysis of Vignette Responses, by Professional Group

Owing to our primary interest in changes to professional judgments to the progressive vignette across its different levels, we focused on whole-sample changes in the main manuscript. As a supplement to Figures 2a-c, we present sub-group scores in this Supplementary Material in Table S7.

Table S7. Judgment outcomes at each stage of the vignette, by professional group

	**Level of disclosure**					
	Attraction		Masturbation		Occupation	
Group →	Primary medical	Mental health	Primary medical	Mental health	Primary medical	Mental health
Competence	2.75 (0.15)	3.68 (0.15)	2.59 (0.16)	3.36 (0.16)	2.57 (0.17)	3.23 (0.17)
Comfort	3.28 (0.16)	3.72 (0.16)	2.84 (0.16)	3.55 (0.16)	2.79 (0.18)	3.27 (0.18)
Need support	5.25 (0.10)	5.08 (0.10)	5.20 (0.11)	5.12 (0.11)	5.47 (0.11)	5.26 (0.11)
Risk judgment	3.40 (0.14)	3.12 (0.14)	3.93 (0.15)	3.78 (0.14)	5.09 (0.14)	4.47 (0.14)
Report (supervisor)	4.76 (0.15)	5.04 (0.14)	4.82 (0.14)	5.12 (0.14)	5.36 (0.12)	5.39 (0.12)
Report (safeguarding)	4.54 (0.16)	3.58 (0.16)	4.70 (0.17)	3.87 (0.16)	5.49 (0.15)	4.74 (0.15)
Report (police)	2.84 (0.15)	2.46 (0.15)	3.12 (0.16)	2.74 (0.16)	3.99 (0.19)	3.50 (0.19)

Note. All outcomes were rated using a 1-6 scale, with high scores indicating more competence, comfort, and desire for support, higher risk judgments, and a greater willingness to report the patient to their supervisor, safeguarding team, or local police force. Data represent estimated marginal means with standard errors presented in parentheses.

Supplementary Materials – Willingness to Treat MAPs (Correlations Table)

In an attempt to streamline the results section of the main manuscript, the editor and reviewers asked us to move non-critical Tables to this Supplementary Material. Below is the table of correlations between the variables used to predict a willingness to treat MAPs.

Table S8. Zero-order correlations within the model predicting willingness to treat MAPs

	1	2	3	4	5	6	7	8	9	10	11	12	13	14
1. Willingness to treat	-													
2. Perceived competence	.39***	-												
3. Anticipated comfort	.49***	.84***	-											
4. Need for more support	-.22**	-.48***	-.46***	-										
5. Desire for more training	-.03	-.37***	-.33***	.45***	-									
6. SPS dangerousness	-.23**	-.21**	-.27**	.11	.15	-								
7. SPS intentionality	-.28*	-.11	-.21**	.02	.04	.46***	-							
8. SPS deviance	-.11	-.28***	-.27**	.16*	.17*	.51***	.34***	-						
9. SPS punitive attitudes	-.22*	-.27**	-.28**	.11	.20*	.61***	.53***	.45***	-					
10. Risk concerns	-.14	-.21*	-.25**	.09	.21***	.43***	.28***	.37***	.50***	-				
11. Knowledge accuracy	.17*	.10	.10	-.07	-.09	-.47***	-.46***	-.28**	-.50***	-.28***	-			
12. Mental health priorities	.29***	.02	.01	.03	.12	-.06	-.23**	.03	-.12	-.07	.09	-		
13. Control priorities	-.05	.10	.09	.00	.08	.21**	.05	.25**	.10	.18*	-.06	.30***	-	
14. Stigma priorities	.24**	.34**	.18*	-.06	.00	-.16*	-.23**	-.26**	-.26**	-.11	.16*	.43***	.24**	-
M	4.22	2.95	3.08	5.14	5.17	4.66	3.37	4.61	3.27	4.54	0.69	8.61	7.47	6.63
(*SD*)	(1.33)	(1.49)	(1.46)	(1.04)	(1.03)	(1.04)	(0.92)	(1.01)	(0.94)	(1.02)	(0.20)	(1.44)	(1.65)	(2.00)
Skewness	-0.85	0.43	0.36	-1.73	-1.83	-0.10	0.57	-0.48	1.26	-0.41	-0.44	-1.06	-0.40	-0.46
Kurtosis	0.18	-0.07	-0.88	3.82	4.19	-0.20	1.12	0.10	2.02	-0.36	0.36	1.00	-0.38	0.16

Note. ‘Priorities’ variables refer to treatment priority factors. SPS = stigma and punitive attitudes scale.

*$p < .05$ **$p < .01$ ***$p < .001$

References

Feldman, D. B., & Crandall, C. S. (2007). Dimensions of mental illness stigma: What about mental illness causes social rejection? *Journal of Social and Clinical Psychology*, *26*(2), 137–154. https://doi.org/10.1521/jscp.2007.26.2.137

Field, A. (2005). *Discovering statistics using SPSS* (2nd ed.). Sage.
